# Single Cell and Spatial Transcriptomics Define a Proinflammatory and Profibrotic Niche After Kidney Injury

**DOI:** 10.1002/advs.202503691

**Published:** 2025-10-03

**Authors:** Li Li, Jinlin Liao, Yuxi Zhang, Zifu Yao, Junxin Huang, Kejia Wu, Lu Li, Yiling Peng, Haili Zhu, Xue Hong, Xi Liu, Lili Zhou, Fan Fan Hou, Haiyan Fu, Youhua Liu

**Affiliations:** ^1^ State Key Laboratory of Multi‐organ Injury Prevention and Treatment National Clinical Research Center of Kidney Disease Division of Nephrology Nanfang Hospital Southern Medical University Guangzhou 510515 China; ^2^ Guangdong Provincial Key Laboratory of Renal Failure Research Guangdong Provincial Institute of Nephrology Guangzhou 510515 China; ^3^ Department of Radiation Oncology Nanfang Hospital Southern Medical University Guangzhou 510515 China

**Keywords:** fibrogenic niche, kidney fibrosis, macrophages, signal cell RNA sequencing, spatial transcriptomics, TNC

## Abstract

Kidney fibrosis is the common outcome of chronic kidney disease (CKD). It often instigates in the focal sites by forming the fibrogenic niche after injury. In this study, using single‐cell RNA sequencing (scRNA‐seq) and a spatial transcriptomic (ST) approach, the cellular heterogeneity, spatial organization, and molecular interactions are delineated in the fibrotic kidney. Through analyses of the scRNA‐seq and ST data from normal and fibrotic kidneys in mice subjected to unilateral ischemia‐reperfusion injury, a tenascin C (TNC)‐enriched, proinflammatory, and profibrotic microenvironment is identified that facilitated macrophage activation and promoted renal inflammation and fibrosis. Both TNC‐enriched decellularized kidney tissue scaffold and exogenous TNC protein promoted bone marrow‐derived macrophages activation though Toll‐like receptor 4 (TLR4)/NF‐κB signaling. Either pharmacological inhibition of TLR4 signaling or genetic knockout of its gene alleviated renal inflammation and fibrosis by inhibiting macrophage activation in vivo. Finally, chimeric mice that received bone marrow transplantation from TLR4‐deficient donors are protected against kidney inflammation and fibrosis. These results suggest that TNC plays a crucial role in orchestrating the formation of a proinflammatory and profibrotic niche that promotes renal inflammation and fibrosis by activating macrophages via TLR4/NF‐κB signaling. The findings underscore the complex interplay among fibroblasts, extracellular microenvironment, and macrophages that drive kidney fibrosis.

## Graphical abstract

By integrating single‐cell RNA sequencing and spatial transcriptomics, tenascin C (TNC) is unveiled as a central organizer of the proinflammatory and profibrotic niche in kidney fibrosis. TNC promotes macrophage activation through TLR4/NF‐κB signaling, leading to renal inflammation and fibrosis. As such, TNC can be a promising therapeutic target for fibrotic kidney disease.

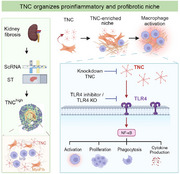

## 1 Introduction

Kidney fibrosis, characterized by excessive accumulation of extracellular matrix (ECM) proteins, stands as a critical pathological hallmark of various chronic kidney diseases (CKD).^[^ ^1^, ^2^ ^]^ Its progression often culminates in end‐stage renal disease, presenting a significant healthcare burden worldwide. The pathological features of kidney fibrosis include the infiltration of inflammatory cells, the extensive activation of interstitial fibroblasts, the dysfunction and atrophy of tubular epithelial cells, the rarefaction and loss of microvasculature, and ultimately the formation of scar tissue leading to the distortion of normal kidney architecture.^[^ ^3^, ^4^, ^5^, ^6^ ^]^ Despite advances in understanding the molecular mechanisms underlying kidney fibrosis, effective therapy to halt or reverse this process remains extremely challenging.^[^ ^3^, ^7^ ^]^

Kidney fibrosis often instigates in distinct focal sites after injury, suggesting that the local tissue microenvironment plays a pivotal role in the initiation and progression of renal fibrotic lesions. We recently proposed the concept of the fibrogenic niche, a specialized microenvironment that facilitates fibroblast activation, ECM production, and scar formation.^[^ ^7^, ^8^ ^]^ Such a niche should contain the cellular components, along with the altered ECM proteins, extracellular vesicles, and soluble molecules that promote the fibrogenesis locally.^[^ ^7^ ^]^ Delineating the cellular heterogeneity, spatial organization, and molecular interactions within the fibrotic niche is a fundamental step in unraveling the mechanisms underlying fibrosis and developing targeted therapeutic strategies.^[^ ^9^, ^10^ ^]^ In this regard, single‐cell RNA sequencing (scRNA‐seq) and spatial transcriptomic (ST) analyses would provide unprecedented insights into the complexities of the fibrotic niche and offer valuable information on the gene expression profiles of individual cells and their spatial distribution.^[^ ^11^, ^12^, ^13^, ^14^ ^]^ By dissecting the fibrotic niche at a single‐cell level and mapping the spatial relationships between different cell types and ECM components, it is conceivable that one can identify key regulatory pathways and potential therapeutic targets, and design interventions that disrupt the fibrotic process and alleviate fibrotic CKD.^[^ ^15^, ^16^ ^]^

Among many elements of the fibrotic niche, the ECM network is of special importance in constructing its spatial organization. As ECM proteins are stationary in nature, they act as the anchor of the fibrotic niche, dictate where the niche assembles by recruiting cellular components and secreted factors, provide structural and biochemical support to surrounding cells, and play a crucial role in modulating their behaviors.^[^ ^7^ ^]^ Of them, tenascin‐C (TNC), a large oligomeric ECM glycoprotein with cell signaling properties,^[^ ^17^ ^]^ is particularly interesting. TNC is often upregulated in fibrotic kidneys and plays a role in organizing the fibrotic niche, orchestrating fibroblast proliferation.^[^ ^18^ ^]^ It also disrupts the integrity of kidney tubular epithelial cells via activating integrin αvβ6 signaling.^[^ ^19^ ^]^ TNC interacts with various ECM components and cell surface receptors, and regulates ECM remodeling, cellular responses, and disease progression.^[^ ^20^ ^]^ However, it remains unclear whether TNC is involved in mediating the cell‐cell interactions among different cell types, such as fibroblasts and immune cells, within the fibrotic niche, thereby contributing to the complex intercellular communication that drives fibrotic nephropathy.

In this study, using an integrated scRNA‐seq and ST approach, we characterized and defined a TNC‐enriched microenvironment as a proinflammatory and profibrotic niche that drives kidney inflammation and fibrosis after injury. We unveiled the intricate cellular and molecular architecture and the spatial immune setting within the fibrotic niche, and provided evidence for coupling renal inflammation and fibrosis via the spatially defined molecular crosstalk between fibroblasts and macrophages. These findings could pave novel way for more precise and effective interventions in combating against kidney fibrosis.

## 2 Results

### 2.1 Systematic Characterization of Spatial Features in Fibrotic Kidneys

**Figure 1:**
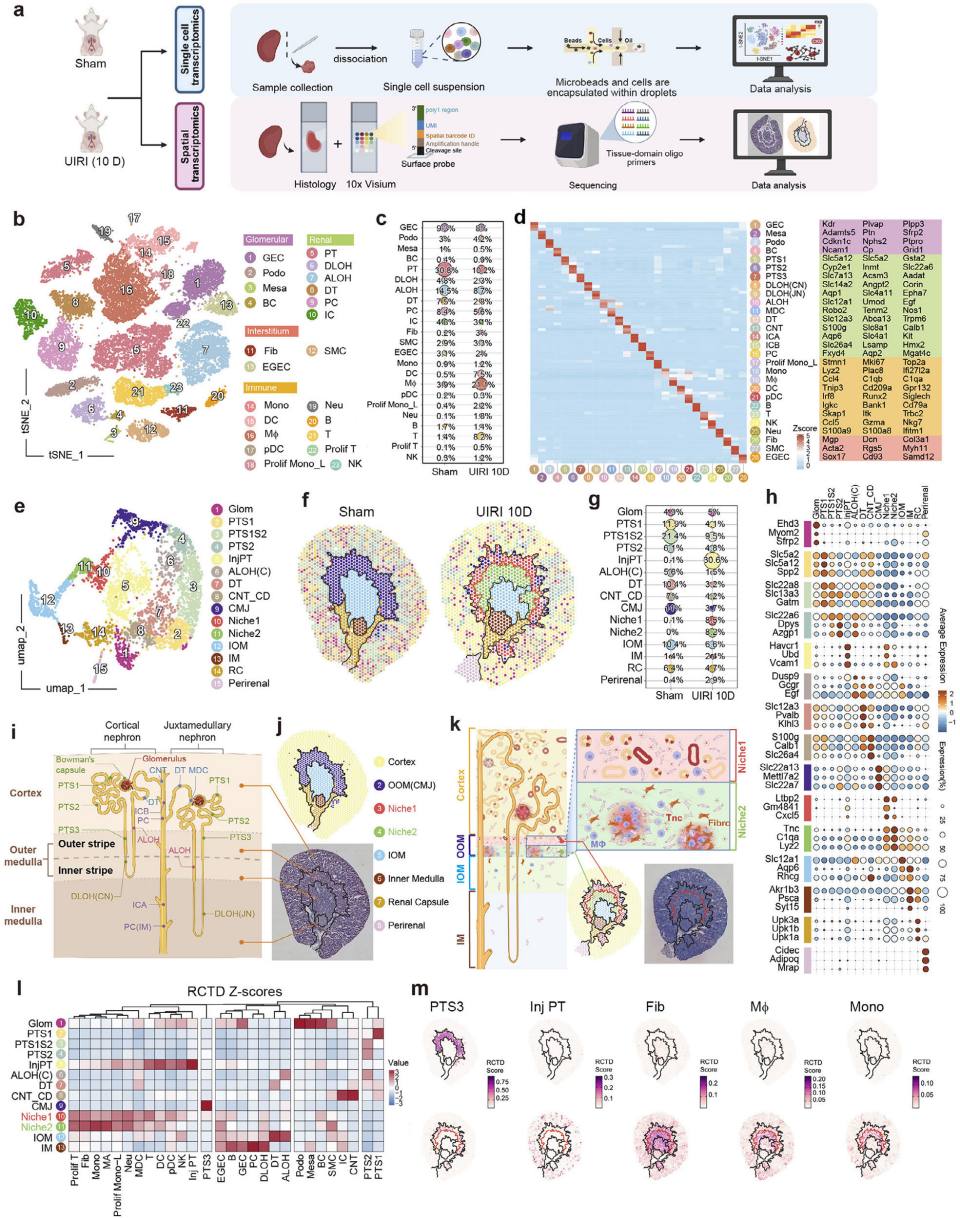
Single‐cell and spatial transcriptome landscape of healthy and fibrotic kidneys after unilateral ischemia‐reperfusion injury (UIRI). a) Schematic representation of single‐cell RNA sequencing (scRNA‐seq) and spatial transcriptomics (ST) of kidneys from the sham and 10‐day UIRI mice, graphically designed with Biorender (https://www.biorender.com/). b) t‐SNE plot illustrating the intricate cellular diversity in fibrotic kidneys, demonstrating distinct clusters representing glomerular endothelial cells (GEC), podocytes (Podo), mesangial cells (Mesa), Bowman's capsule epithelium (BC), proximal tubules (PT), descending limbs of Henle (DLOH), ascending limbs of Henle (ALOH), distal tubules (DT), principal cells (PC), intercalated cells (IC), fibroblasts (Fib), smooth muscle cells (SMC), extraglomerular endothelial cells (EGEC), monocytes (Mono), dendritic cells (DC), macrophages (Mϕ), plasmacytoid dendritic cells (pDC), proliferating mononuclear lineage (Prolif mono_L), and neutrophils (Neu), B cells (B), T cells (T), proliferating T cells (prolif T), and natural killer cells (NK). These cell types were further categorized into four major compartments: Glomerular, Renal, Interstitium, and Immune, as indicated by color grouping in the plot. c) Bubble plot illustrating the relative proportions of major kidney cell types in sham and UIRI samples. Each dot represents the proportion of a given cell type in a specific sample group, with dot size corresponding to its relative proportion. d) A comprehensive heatmap depicting the unique marker gene signature of major renal cell types. e) UMAP plot illustrating the inferred renal cell region distribution based on integrated spatial transcriptomics data from normal (Sham) and UIRI 10D mouse kidneys, generated using the 10x Genomics Visium platform. The identified regions include glomerular cells (Glom), distinct segments of the proximal tubule (PTS1, PTS1S2, PTS2), injured proximal tubules (InjPT), ascending limbs of Henle in cortex (ALOH(C)), distal tubules (DT), connecting tubules and collecting ducts (CNT_CD), cells at the corticomedullary junction (CMJ), fibrogenic niche regions (Niche1, Niche2), the inner stripe of the outer medulla (IOM), inner medulla (IM), renal capsule (RC), and perirenal tissue (Perirenal). f) Spatial maps illustrating the anatomical distribution of renal cell regions in Sham and UIRI 10D mouse kidneys. Region colors correspond to the classifications defined in panel (e). g) Bubble plot illustrating the relative proportions of major renal cell regions in spatial transcriptomics data from sham and UIRI 10D mouse kidneys. h) Bubble plot depicting the expression patterns of marker genes across distinct renal cell regions in spatial transcriptomics data. Dot color indicates the average gene expression level within each region, while dot size represents the proportion of spatial spots expressing the gene. i) Schematic diagram of nephron segmentation by cell types. j) Comparison of kidney anatomical regions and spatial transcriptomic clusters, showing clusters in kidney tissue (top) and the corresponding Visium H&E‐stained section (bottom). k) Renal tissue structure alterations at the corticomedullary junction (CMJ) in UIRI samples, showing the formation of two distinct fibrogenic niches, Niche1 and Niche2. l) A heatmap showing the deconvolution scores of cell type compositions across different regions in Visium spatial transcriptomics data, obtained using the RCTD method. m) Spatial FeaturePlots of RCTD‐derived cell type scores in the sham (top) and UIRI (bottom) groups, with paired panels sharing a common legend.

To investigate the cellular and molecular composition of the fibrogenic niche in CKD, we performed single‐cell RNA sequencing (scRNA‐seq) and 10x Visium spatial transcriptomics to systematically compare kidney cell composition and molecular profiles between sham‐operated (sham) and 10‐day unilateral ischemia‐reperfusion injury (UIRI 10D) mice (**Figure** **1**a). Using classical marker genes,^[^ ^21^, ^22^, ^23^, ^24^ ^]^ we identified major kidney cell types (Figure 1b; Figure S1b–f, Supporting Information) and found that fibrotic kidneys exhibited a significant increase in injured tubular epithelial cells, immune‐infiltrating cells, and fibroblasts (Figure 1c). Heatmap analysis further revealed the characteristic gene expression signatures of each cell subset (Figure 1d). Cross‐referencing with published kidney single‐cell datasets confirmed the reliability of our annotations (Figure S1i–k, Supporting Information).

Integration of Visium data from sham and UIRI groups revealed dramatic spatial reorganization (Figure 1e–h). In sham kidneys, the expected corticomedullary zonation was preserved, encompassing the cortex, outer stripe of outer medulla (OOM, also referred to as the corticomedullary junction [CMJ]), inner stripe of outer medulla (IOM), and inner medulla (IM) (Figure 1i,j). In contrast, UIRI 10D kidneys displayed marked injury‐associated tubular enrichment in the cortex (Figure S1g,h, Supporting Information), with the most profound remodeling occurring at the CMJ, in which the typical PTS3 tubular segment, normally localized to OOM, was replaced by immune cell and fibroblast infiltration, forming two distinct fibrogenic niches (Niche1 and Niche2) (Figure 1k; Figure S2a,h, Supporting Information). RCTD‐based deconvolution of spatial data showed that Niche1 consisted of fibroblasts, immune cells, and residual tubular cells, while Niche2 featured specialized fibroblast‐macrophage/monocyte co‐localization (Figure 1l,m; Figure S2h‐left, Supporting Information).^[^ ^25^ ^]^ Notably, although fibroblasts infiltrated into the IOM, only the CMJ sustained stable fibroblast‐macrophage interactions, suggesting this unique spatial architecture may represent a key microenvironment driving fibrosis.

### 2.2 High‐Resolution Spatial Transcriptomics Unveils Molecular Signatures of Fibrogenic Niches

**Figure 2:**
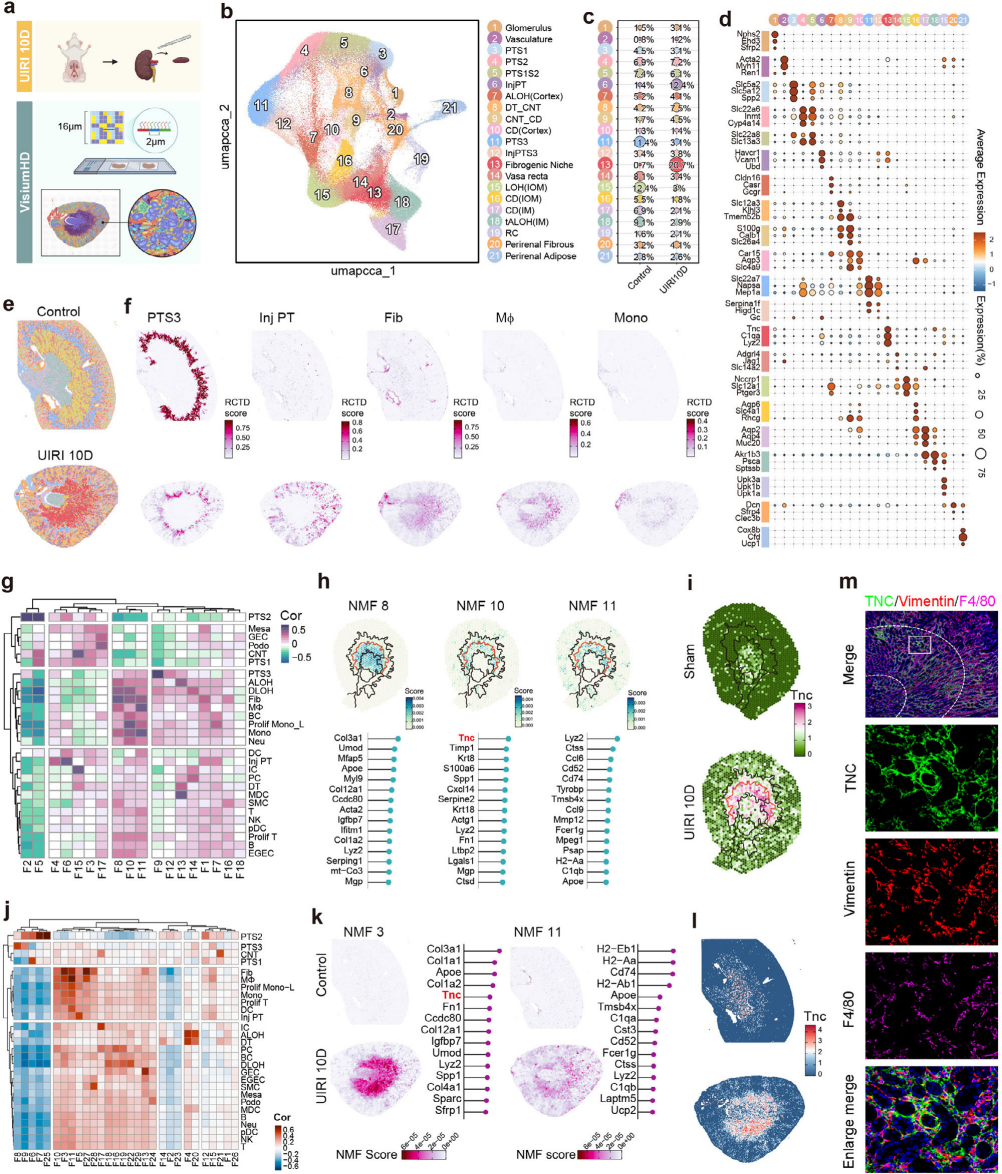
High‐resolution spatial transcriptomics and immunostaining reveal the TNC‐enriched fibroblast‐macrophage niche organization in fibrotic kidneys. a) Schematic diagram of the Visium HD workflow applied to kidney tissues from sham and UIRI model mice. b) UMAP visualization of integrated Visium HD spatial transcriptomics data from control mice (obtained from the 10x Genomics public dataset) and UIRI mice (this study), processed using canonical correlation analysis (CCA). This dimensionality reduction visualization reveals distinct clusters representing various renal parenchymal and stromal cell populations, including: Glomerulus, Vasculature, PTS1, PTS2, PTS1S2, InjPT, ascending limbs of Henle in cortex [ALOH(Cortex)], distal tubule and connecting tubule (DT_CNT), connecting tubule and collecting duct (CNT_CD), collecting duct in cortex [CD(Cortex)], PTS3, injured PTS3 (InjPTS3), Fibrogenic Niche, Vasa recta, loop of Henle in outer medulla [LOH(IOM)], collecting duct in outer medulla [CD(IOM)], collecting duct in inner medulla [CD(IM)], thin ascending limbs of Henle in inner medulla [tALOH(IM)], renal capsule (RC), Perirenal Fibrous tissue, and Perirenal Adipose tissue. c) Bubble plot comparing the regional distribution in Control versus UIRI 10d kidneys (Visium HD). d) Bubble plot depicting the expression patterns of marker genes across distinct renal cell regions in Visium HD data. e) Spatial maps generated using Visium HD illustrate the inferred anatomical distribution of renal cell regions in kidney tissues from Control and UIRI mice. f) Spatial Feature Plots of Visium HD data showing the spatial distribution of selected renal cell types in controls (top) and UIRI mice (bottom), based on cell‐type deconvolution using RCTD. g) A heatmap showing the correlation between NMF factors and cell‐type deconvolution scores in standard Visium spatial transcriptomics data. h) Spatial distribution of gene scores associated with the NMF factors most correlated with the fibrogenic niche, along with the contribution of key genes to each factor. i) Spatial FeaturePlots showing the anatomical distribution of Tnc expression in standard Visium. j) A heatmap showing the correlation between NMF factors and cell type deconvolution scores in Visium HD spatial transcriptomics data. k) Spatial distribution of NMF factors (NMF3 and NMF11) associated with the fibrogenic niche in Visium HD data, along with their corresponding high‐contributing genes. l) Spatial FeaturePlots showing the anatomical distribution of Tnc expression in Visium HD datasets. m) Immunofluorescence staining demonstrates colocalization of TNC with macrophages (F4/80⁺) in the CMJ interstitial region. From top to bottom: an overview merged image (Merge), followed by magnified views of TNC, Vimentin, and F4/80 staining in the same region, and an enlarged merged image (Enlarged Merge) at the bottom.

To dissect the molecular underpinnings of fibrogenic niches, we conducted ultra‐high‐resolution Visium HD analysis on UIRI 10D and control kidneys (**Figure** **2**a–c; Figure S2c, Supporting Information). Compared to conventional Visium, Visium HD not only recapitulated cortical tubular injury and CMJ fibroblast infiltration but also precisely demarcated fibroblast‐macrophage clusters with sharp spatial boundaries (Figure 2d–f; Figure S2f‐right, Supporting Information).

Non‐negative matrix factorization (NMF) of Visium data identified spatially variable gene modules: Factor 8 (collagen‐related genes, broadly distributed in fibroblast‐infiltrated regions) and Factors 10/11 (selectively enriched in fibrogenic niches) represented distinct biological features (Figure 2g; Figure S2b, Supporting Information). Factor 11 reflected macrophage‐specific genes, while tenascin‐C (Tnc), a core component of Factor 10, exhibited strong co‐localization with fibroblast‐macrophage niches (Figure 2h). Visium HD‐based NMF further validated these findings (Factor 3: fibroblast‐associated; Factor 11: macrophage‐associated; Figure 2i–l; Figure S2d, Supporting Information). Single‐cell lineage tracing confirmed that Tnc was primarily secreted by fibroblasts (Figure S2e, Supporting Information), and its spatial expression was strictly confined to CMJ fibroblast‐macrophage clusters, absent from fibroblast‐invaded IOM regions (Figure 2i–l). Cell‐cell interaction analysis (excluding fibroblast autocrine signals) revealed that the top 10 molecules co‐expressed with Tnc were macrophage‐derived (Figure S2f,g, Supporting Information). Immunofluorescence verified TNC protein deposition in CMJ interstitial regions, co‐localizing with F4/80+ macrophages (Figure 2m). Collectively, these findings demonstrate that fibroblasts establish a molecular framework for fibrogenic niches via spatially restricted secretion of TNC.

### 2.3 TNC‐Enriched Niche Promotes Macrophages Activation

**Figure 3:**
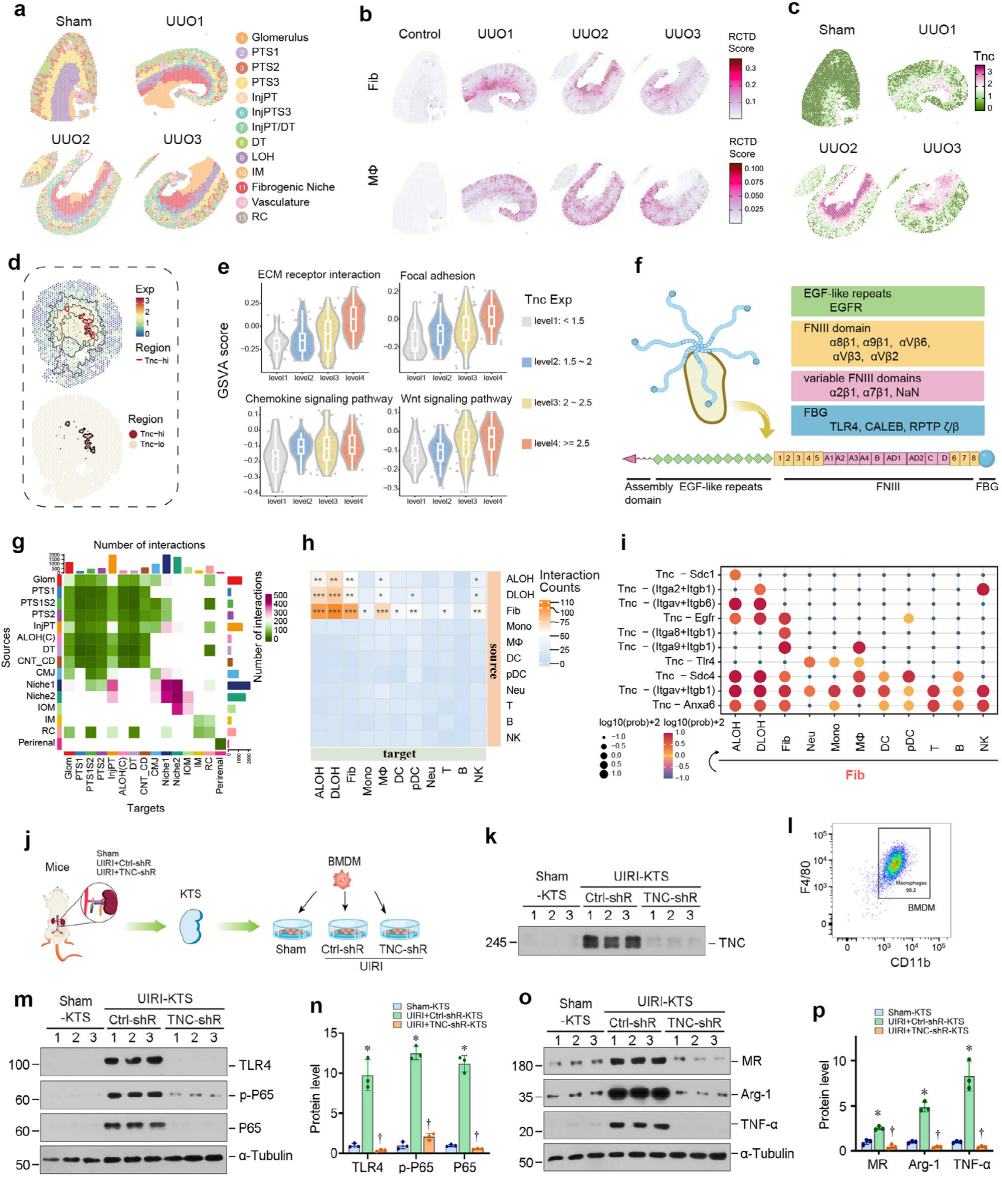
TNC‐enriched microenvironment promotes macrophages proliferation and activation. a) Spatial maps illustrating the anatomical distribution of inferred renal cell regions in sham and unilateral ureteral obstruction (UUO) models. b) Spatial Feature Plots illustrating RCTD‐derived scores for Fib and Mϕ cell types in the sham and UUO groups. c) Spatial map showing the expression pattern of the Tnc gene in the sham and UUO samples. d) Spatial expression profile of Tnc in fibrotic kidneys is illustrated (top). The red color spots represent areas with high Tnc expression (Exp ≥ 2.5) (bottom). e) Spots from the UIRI group were categorized into four levels by Tnc expression. Differential pathway analysis was performed using limma (P value ≤ 0.1) and visualized using violin plots. f) Schematic diagram displaying the structural domains of Tnc and corresponding receptors. g) CellChat‐predicted ligand‐receptor interaction heatmap across spatial regions in UIRI groups, with color intensity representing the number of significant interaction pairs. h) The heatmap depicts the number of cellular interactions in fibroblast‐enriched areas of the UIRI group. Color intensity correlates with the count of ligand‐receptor pairs. Sender specifies the signaling source and target the signal recipient. “Sender” refers to the signal source, and “target” denotes the signal recipient. i) The bubble chart depicts fibroblast interactions with various cell types through Tnc, with color depth and bubble size reflecting log10 (Prob+1). “Prob” indicates interaction probability, with a threshold of 0.01 j) Diagram showed the experimental protocol by which BMDMs were cultured on TNC‐rich or ‐depleted kidney tissue scaffold (KTS). The KTS was prepared from sham, UIRI+Ctrl‐shRNA, and UIRI+TNC‐shRNA kidneys in mice. BMDMs were inoculated on different KTS for 48 h. k) Knockdown of TNC in mice resulted in its depletion in the KTS. Representative Western blot analyses show TNC protein levels in the KTS prepared from different groups as indicated. l) Flow cytometry analysis shows the purity of BMDMs by CD11b and F4/80 staining. The percentage of CD11b ^+^ F4/80 ^+^ population is > 95%. m,n) TNC‐rich KTS induces TLR4, p‐P65 and P65 expression. Western blots analyses show the expression of TLR4, p‐P65, and P65 in different groups as indicated. Representative Western blot (m) and quantitative data (n) are shown. ^*^ *p* < 0.05 versus sham, ^†^ *P* < 0.05 versus UIRI+Ctrl‐shRNA (n = 3). o,p) TNC‐rich KTS induces MR, Arg‐1, and TNF‐α expression in BMDM. Western blots analyses show the expression of MR, Arg‐1, and TNF‐α in different groups as indicated. Representative Western blot (o) and quantitative data (p) are shown. ^*^ *p* < 0.05 versus sham, ^†^ *P* < 0.05 versus UIRI+Ctrl‐shRNA (n = 3).

We also analyzed single‐cell and spatial transcriptomic data from unilateral ureteral obstruction (UUO) models (**Figure** **3**a; Figures S3 and S4, Supporting Information). Although UUO‐induced fibrosis primarily localized to the IOM‐IM junction, which was distinct from UIRI's CMJ predilection, this region similarly exhibited fibroblast‐macrophage co‐clustering and conserved TNC spatial patterning (Figure 3b,c; Figure S4a–c). GSVA showed that escalating TNC expression correlated with ECM remodeling, cell adhesion, and Wnt pathway activation (Figure 3d–e), consistent with our prior work.^[^ ^20^ ^]^

Spatial ligand‐receptor analysis identified a prominent fibroblast‐macrophage communication hub in the CMJ (Figure 3g).^[^ ^26^ ^]^ Single‐cell interaction prediction suggested that fibroblast‐derived TNC engages macrophage TLR4 via its fibrinogen‐like globular (FBG) domain^[^ ^17^, ^27^, ^28^, ^29^ ^]^ (Figure 3f,h,I; Figure S4d,e and Table S4, Supporting Information), offering mechanistic insight into matrix‐immune crosstalk within fibrogenic niches.

To experimentally validate the role of TNC in mediating the interaction between fibroblasts and macrophages, we examined the impact of the TNC‐enriched kidney tissue microenvironment on macrophage biology using an ex vivo model system. Figure 3j shows the experimental protocol. Specifically, we performed in vivo experiments utilizing a short hairpin RNA (shRNA)‐mediated knockdown of endogenous TNC.^[^ ^19^ ^]^ Specifically, mice were injected intravenously with either control‐shRNA or TNC‐shRNA plasmid vectors at 4 days after UIRI (Figure 3j). We then conducted ex vivo studies using decellularized kidney tissue scaffold (dc‐KTS) by inoculating bone marrow‐derived macrophages (BMDMs) onto them (Figure 3j). Knockdown of TNC in vivo resulted in its deficiency in the dc‐KTS of UIRI mice (Figure 3k). BMDMs were cultured and characterized, with the purity of the CD11b ^+^ F4/80^ +^ population exceeding 95%, as illustrated in Figure 3l. As shown in Figure 3m–p, when BMDMs were cultivated in the TNC‐rich dc‐KTS from UIRI mice, TLR4, p‐P65, P65, mannose receptor (MR), arginase‐1 (Arg‐1), and tumor necrosis factor‐α (TNF‐α) were dramatically induced. However, depletion of TNC in UIRI‐KTS abolished these inductions. Together, these results unambiguously demonstrate that TNC‐enriched microenvironment promotes macrophage activation.

### 2.4 TNC Promotes Macrophage Proliferation and Activation In Vitro

**Figure 4:**
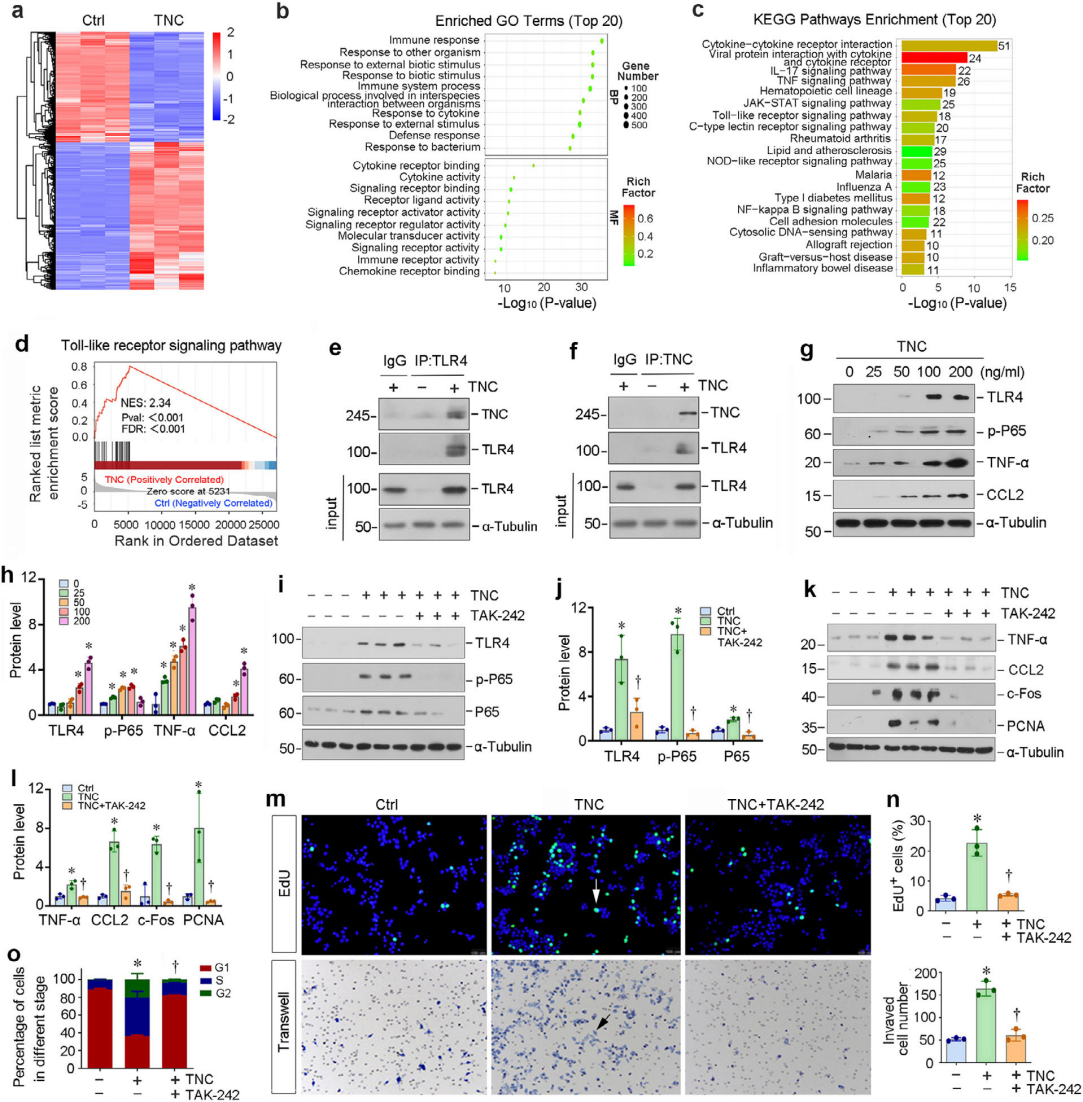
TNC induces macrophages proliferation and activation by triggering the TLR4/NF‐κB signaling cascade in vitro. a) The mRNA clustering showed significant differences in gene expression between the control group and TNC stimulated macrophages for 24 h. b) GO enrichment analysis shows that several signaling pathways as indicated were enriched. c) KEGG enrichment analysis reveals that several signaling pathways were enriched. d) GSEA enrichment analysis shows that Toll‐like receptor (TLR) signaling pathway was enriched in TNC‐stimulated macrophages. NES, normalized enrichment score; FDR, false discovery rate. e,f) Co‐immunoprecipitation (Co‐IP) demonstrated that TNC bound to TLR4 in macrophages. BMDMs were incubated in the absence or presence of TNC. Cell lysates were immunoprecipitated with IgG, anti‐TLR4 or anti‐TNC, followed by immunoblotting with anti‐TNC and anti‐TLR4. g,h) TNC activated TLR4/NF‐κB signal cascade in vitro. BMDMs were treated with different concentrations of TNC as indicated for 48 h. Representative Western blot (g) and quantitative data (h) are shown. ^*^ *p* < 0.05 versus Ctrl, ^†^ *P* < 0.05 versus TNC (n = 3). i,j) Blockade of TLR4 inhibited NF‐κB signal activation induced by TNC in BMDMs. BMDMs were pretreated with TLR4 inhibitor TAK‐242 (5 nM) for 2 h, then treated with TNC (100 µg ml^−1^) for 48 h. Western blots (i) and quantitative data (j) are presented. ^*^ *p* < 0.05 versus Ctrl, ^†^ *P* < 0.05 versus TNC (n = 3). k,l) Blockade of TLR4/NF‐κB signaling abolished TNC‐induced macrophage proliferation and activation. BMDMs were pretreated with TLR4 inhibitor TAK‐242 (5 nM) for 2 h, then treated with TNC (100 µg ml^−1^) for 48 h. Western blots (k) and quantitative data (l) are presented. ^*^ *p* < 0.05 versus Ctrl, ^†^ *P* < 0.05 versus TNC (n = 3). m,n) Blocking TLR4 inhibited TNC‐induced macrophage proliferation and migration. EdU incorporation assay shows that TLR4 inhibitor TAK‐242 inhibited TNC‐induced cell DNA synthesis in BMDMs. Representative EdU incorporation assay (m) and quantitative data (n) are shown. The Transwell assay also showed that blocking TLR4 inhibited TNC‐induced macrophage migration. Representative transwell migration assay (m) and quantitative data (n) are presented. ^*^ *p* < 0.05 versus Ctrl, ^†^ *P* < 0.05 versus TNC (n = 3). o) Flow cytometry shows the distribution of different phases in the cell cycle in BMDMs after incubation with TNC (100 ng ml^−1^) for 48 h. Quantitative data (m) is shown. ^*^ *p* < 0.05 versus Ctrl, ^†^ *P* < 0.05 versus TNC (n = 3).

To further confirm the role of TNC in mediating macrophage activation, we conducted in vitro experiments by directly incubating BMDMs with human TNC protein. We then employed the RNA‐seq approach to investigate the gene expression profile of macrophages in different groups. As shown in **Figure** **4**a, the mRNA clustering showed significant differences in gene expression between the control and TNC‐stimulated macrophages. Furthermore, the enrichment analysis of Gene Ontology terms revealed abundant genes associated with immune response, cytokine receptor binding, and immune receptor activity (Figure 4b). KEGG analysis revealed that several inflammation‐related signaling pathways were enriched, including cytokine‐receptor interaction, TNF, TLR, and NF‐κB signaling (Figure 4c).

We next examined the effect of TNC on macrophage proliferation, activation, cytokine production, migration, and phagocytosis. As shown in Figure S5a,b (Supporting Information), TNC upregulated proliferating cell nuclear antigen (PCNA) and c‐Fos expression in BMDMs. Furthermore, TNC promoted the expression of macrophage activation markers, inducible nitric oxide synthase (iNOS) and TNF‐α (Figure S5a,b, Supporting Information). Similar results were obtained by the EdU incorporation assay. As shown in Figure S5c,d (Supporting Information), TNC promoted EdU incorporation in macrophages, indicating its ability to promote cell cycle entry and subsequent DNA synthesis. Further cell cycle analysis by flow cytometry revealed that TNC induced an increased percentage of cells in G2/M and S phase (Figure S5e–g, Supporting Information). Flow cytometry detected an increased phagocytosing GFP fluorescent microspheres by macrophages after TNC incubation (Figure S5h,i, Supporting Information). In addition, TNC increased the migration of macrophages (Figure S5j,k, Supporting Information). This series of data indicate that TNC promotes macrophage proliferation, activation, migration, and phagocytosis in vitro. To assess the specificity of TNC in promoting macrophage activation within a broader ECM milieu, we performed systematic comparative analyses using recombinant collagen I and fibronectin, two abundant ECM proteins at concentrations matching or exceeding TNC doses. Unlike TNC, neither collagen I nor fibronectin affected the phenotypic markers of BMDMs, as demonstrated by unchanged protein levels of TNF‐α and C‐C motif chemokine ligand 2 (CCL2), also known as monocyte chemoattractant protein‐1 (MCP‐1), c‐Fos, and PCNA (Figure S5l–o, Supporting Information). These data collectively indicate that TNC specifically promotes macrophage activation, proliferation, migration, phagocytosis, and cytokine production.

### 2.5 TNC Activates Macrophages via TLR4/NF‐κB Signaling

We found that TLR signaling was enriched in TNC‐stimulated macrophages (Figure 4d), which is consistent with previous reports that TNC, as a type of damage‐associated molecular patterns (DAMPs), can bind to cell surface pattern recognition receptors.^[^ ^30^, ^31^ ^]^ To determine whether TNC directly binds to TLR4, we performed co‐immunoprecipitation (Co‐IP) assay. As shown in Figure 4e,f, TNC and TLR4 were reciprocally detected in the immunoprecipitates using either anti‐TNC or anti‐TLR4 antibody, confirming their physical interaction. We further stimulated macrophages with TNC in different concentrations in vitro. As shown in Figure 4g,h, Western blotting showed that TNC upregulated TLR4, p‐P65, TNF‐α, and CCL2 expression in a dose‐dependent manner, indicating that TNC activates the TLR4/NF‐κB signaling.

We next used the TLR4 specific small molecule inhibitor TAK‐242, which can competitively bind to the intracellular region of TLR4,^[^ ^32^ ^]^ to assess whether it blocks TNC‐mediated macrophage activation. As shown in Figure 4i–l, TAK‐242 effectively abolished TLR4 induction and P65 activation by TNC and inhibited the expressions of TNF‐α, CCL2, c‐Fos, and PCNA proteins, underscoring that TLR4 signaling is necessary for TNC‐mediated macrophage proliferation and activation. Consistently, EdU staining showed that blocking TLR4 repressed TNC‐induced DNA synthesis in BMDMs (Figure 4m,n). The Transwell assay also showed that TAK‐242 inhibited TNC‐induced macrophage migration (Figure 4m,n). In addition, flow cytometry analysis revealed that blockade of TLR4 signaling abolished macrophages to enter into the S phase (Figure 4o).

To further validate the functional role of TLR4 in TNC‐mediated macrophage activation, we stimulated BMDMs with TNC in the presence or absence of a TLR4 blocking antibody (TLR4‐blo Ab). As shown in Figure S6a–d (Supporting Information), TLR4‐blo Ab suppressed TNC‐induced TLR4/NF‐κB signaling. Consistently, the induction of TNF‐α, CCL2, c‐Fos, and PCNA proteins triggered by TNC was also abolished. Together, these results suggest that TNC activates macrophages by stimulating TLR4/NF‐κB signal cascade.

### 2.6 Endogenous TNC Aggravates Renal Inflammation In Vivo

**Figure 5:**
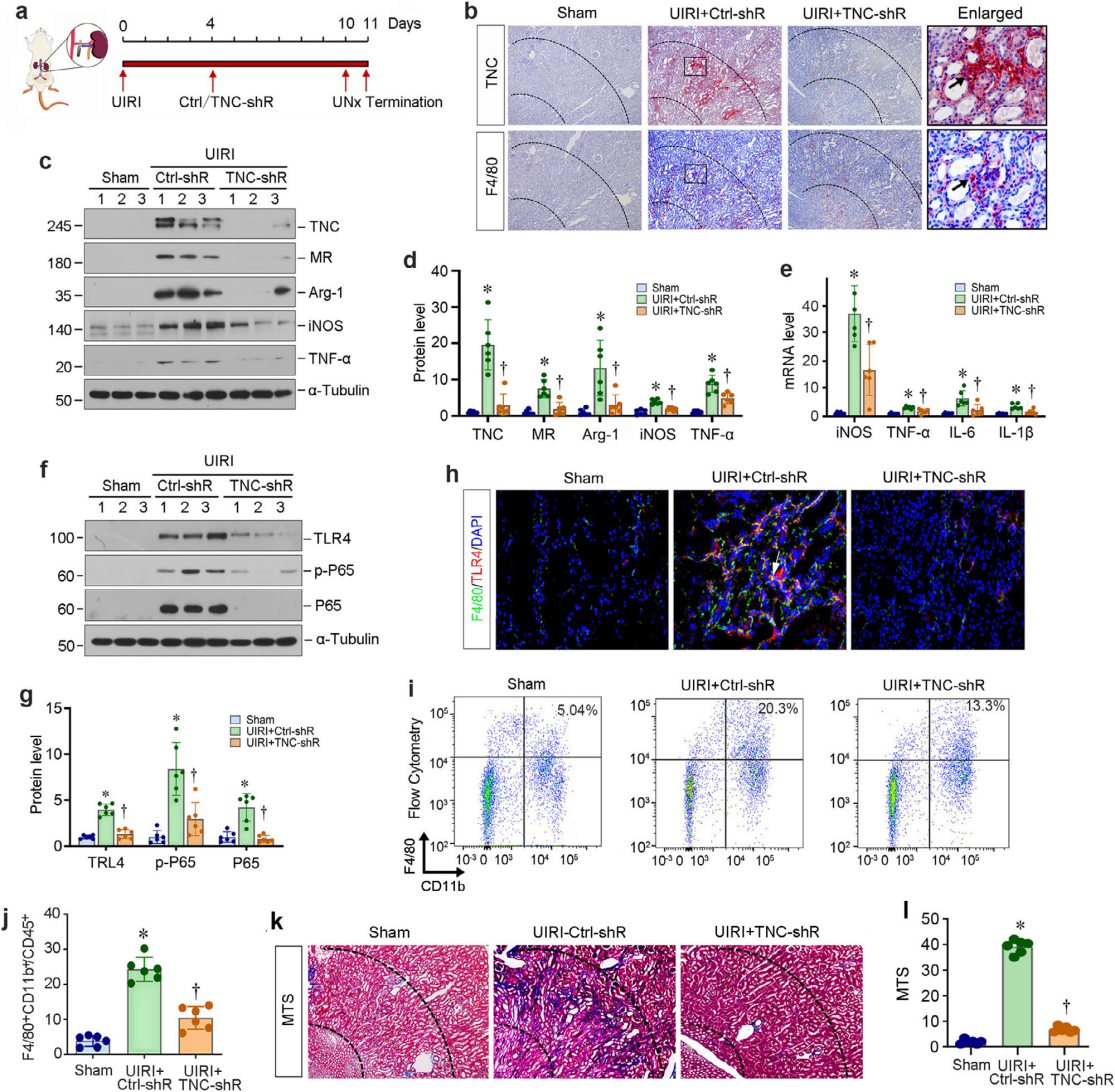
Knockdown of TNC ameliorates renal inflammation after UIRI. a) The diagram shows the experimental protocol. Knockdown of TNC was carried out by a short hairpin RNA (shRNA)‐mediated approach. Mice were injected intravenously with either control‐shRNA or TNC‐shRNA plasmids at 4 days after UIRI. Red arrows indicate the different timing of UIRI, injecting plasmids, uninephrectomy (UNx) or sacrifice. b) Immunohistochemical staining on adjacent serial sections showed co‐localization of TNC and F4/80. Arrowheads indicate TNC and F4/80 positive cells. The areas between the dashed lines represent the corticomedullary junction of the kidney. c,d) Western blot analyses show renal expression of TNC, MR, Arg‐1, iNOS, and TNF‐α proteins in different groups as indicated. Representative Western blot (c) and quantitative data (d) are shown. ^*^ *p* < 0.05 versus Sham, ^†^ *P* < 0.05 versus UIRI + Ctrl‐shRNA (n = 6). e) qRT‐PCR analyses show knockdown of TNC inhibited renal mRNA levels of iNOS, TNF‐α, IL‐6, and IL‐1β. ^*^ *p* < 0.05 versus Sham, ^†^ *P* < 0.05 versus UIRI + Ctrl‐shR (n = 6). f,g) Western blot analyses show renal expression of TLR4, p‐P65, and P65 proteins in different groups as indicated. Representative Western blot (f) and quantitative data (g) are shown. ^*^ *p* < 0.05 versus sham, ^†^ *P* < 0.05 versus UIRI + Ctrl‐shRNA (n = 6). h) Representative immunofluorescence images of F4/80 and TLR4 in the renal corticomedullary junction area in different groups as indicated. i,j) Flow cytometry shows the distribution of F4/80^+^/CD11b^+^/CD45^+^ macrophages in different groups as indicated. Representative flow cytometry histograms (i) and quantitative data (j) are shown. ^*^ *p* < 0.05 versus Sham, ^†^ *P* < 0.05 versus UIRI + Ctrl‐shR (n = 6). k,l) Knockdown of TNC ameliorated the fibrotic lesions of the kidneys after UIRI. (k) Representative micrographs show collagens deposition by Masson's trichrome staining (MTS) in different groups as indicated. The areas between the dashed lines represent the corticomedullary junction of the kidney. (l) Graphic presentation showed the semi‐quantitative determination of fibrotic lesions in different groups. At least 10 randomly selected fields were assessed, and the results averaged for each kidney. ^*^ *p* < 0.05 versus Sham, ^†^ *P* < 0.05 versus UIRI + Ctrl‐shR (n = 6).

To elucidate the role of TNC in mediating macrophage activation in vivo, we performed animal experiments utilizing a short hairpin RNA (shRNA)‐mediated approach to knock down TNC.^[^ ^19^, ^33^ ^]^ Specifically, mice were injected intravenously with either control‐shRNA or TNC‐shRNA plasmid vectors at 4 days after UIRI (**Figure** **5**a). As illustrated in Figure 5b, immunohistochemical staining showed that in the sites where TNC protein expression was upregulated and focally distributed, a significant increase in F4/80^+^ macrophages was observed. When the TNC in the renal interstitium was reduced after knocking down its expression, infiltration of F4/80^+^ macrophages was also consistently decreased at the same location (Figure 5b).

We further examined the effect of TNC depletion on macrophage activation after UIRI in vivo. As illustrated in Figure 5c,d, knockdown of TNC inhibited renal expressions of MR, Arg‐1, iNOS, and TNF‐α after UIRI. Similarly, knockdown of TNC also inhibited renal mRNA levels of iNOS, TNF‐α, IL‐6, and IL‐1β (Figure 5e). Knockdown of TNC inhibited protein expressions of TLR4, p‐P65, and P65 (Figure 5f,g). Immunofluorescence staining revealed that F4/80^+^ and TLR4^+^ cells were increased and co‐localized in the renal CMJ region of UIRI mice, compared to the controls (Figure 5h). After knocking down TNC, F4/80^+^/TLR4^+^ macrophages were markedly decreased (Figure 5h). To quantitatively detect the subpopulations of renal macrophages, single‐cell suspensions were prepared from the control or UIRI kidneys. As shown in Figure 5i,j, compared with the sham group, a higher number of F4/80^+^/CD11b^+^/CD45^+^ macrophages was detected in the kidneys of UIRI mice, which was reduced after TNC depletion. In addition, Masson's trichrome staining (MTS) showed that knockdown of TNC attenuated renal fibrotic lesions in the CMJ area of the UIRI kidney (Figure 5k,l). These results suggest that endogenous TNC promotes macrophage activation and renal fibrosis.

### 2.7 Inhibition or Knockout of TLR4 Prevents Renal Inflammation and Fibrosis

**Figure 6:**
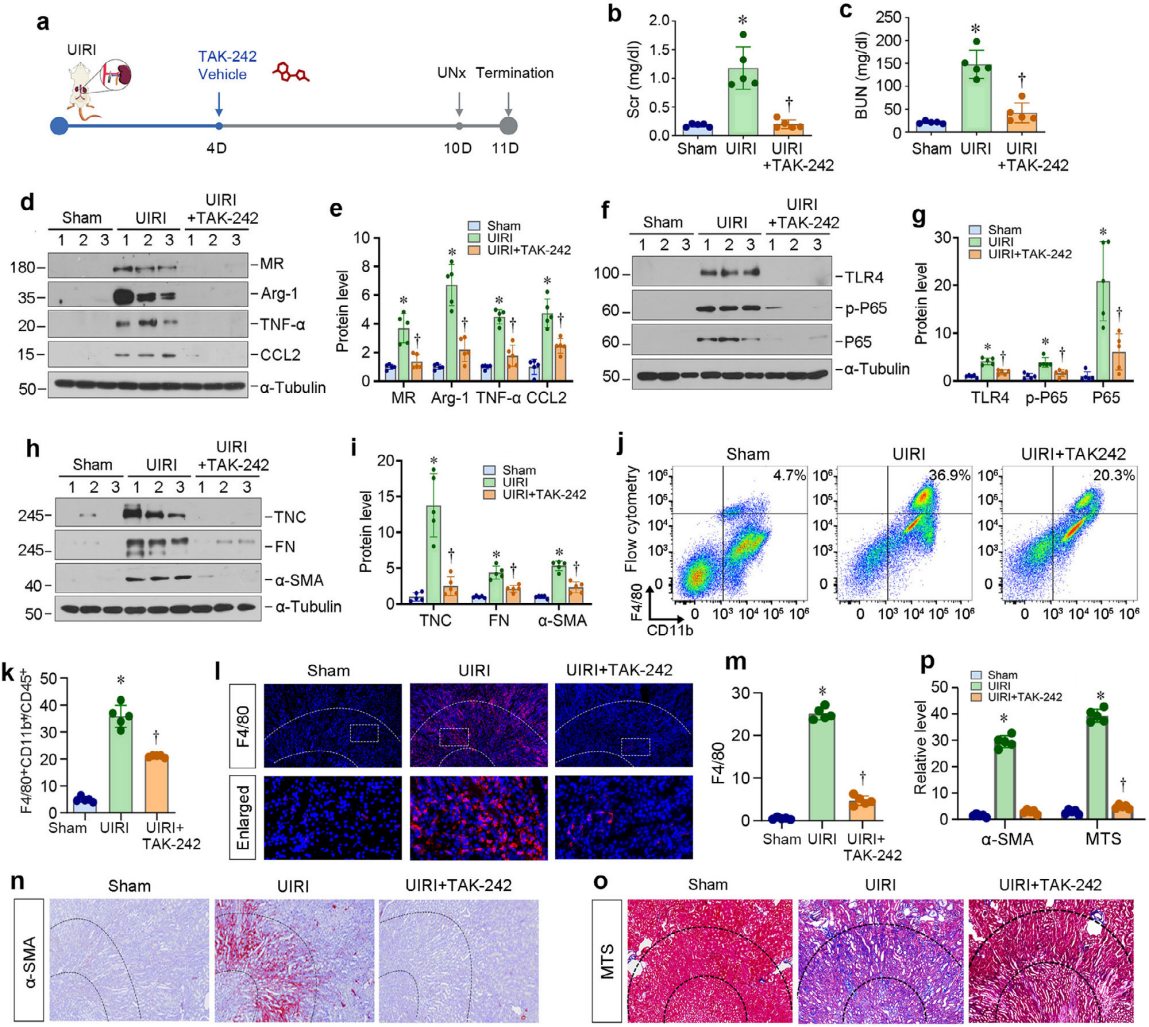
Inhibition of TLR4 attenuates renal inflammation and renal fibrosis in vivo. a) The diagram shows the experimental protocol. Round dots indicate the different timing of UIRI, intraperitoneal injection with TAK‐242 (8 mg kg^−1^) or vehicle, uninephrectomy (UNx) or sacrifice. b,c) Graphic presentations show serum creatinine (Scr) (b) and blood urea nitrogen (BUN) (c) levels in different groups as indicated. ^*^ *p* < 0.05 versus sham, ^†^ *P* < 0.05 versus UIRI (n = 5). d,e) Western blot analyses show renal expression of MR, Arg‐1, TNF‐α, and CCL2 in different groups as indicated. Representative Western blot (d) and quantitative data (e) are shown. ^*^ *p* < 0.05 versus sham, ^†^ *P* < 0.05 versus UIRI (n = 5). f,g) Western blot analyses show renal expression of TLR4, p‐P65 and P65 in different groups as indicated. Representative Western blot (f) and quantitative data (g) are shown. ^*^ *p* < 0.05 versus sham, ^†^ *P* < 0.05 versus UIRI (n = 5). h,i) Western blot analyses show renal expression of TNC, FN, and α‐SMA in different groups as indicated. Representative Western blot (h) and quantitative data (i) are shown. ^*^ *p* < 0.05 versus sham, ^†^ *P* < 0.05 versus UIRI (n = 5). j,k) Inhibition of TLR4 ameliorated renal inflammation in vivo. Flow cytometry showed the distribution of F4/80^+^/CD11b^+^/CD45^+^ macrophages in different groups as indicated. Representative flow cytometry histograms (j) and quantitative data (k) are shown. ^*^ *p* < 0.05 versus sham, ^†^ *P* < 0.05 versus UIRI (n = 5). l,m) Inhibition of TLR4 reduces renal inflammation in vivo. Representative micrographs (l) show renal expression and localization of F4/80 by immunofluorescence staining in different groups as indicated. The areas between the dashed lines represent the corticomedullary junction of the kidney. Graphic presentation (m) shows the semi‐quantitative determination of renal F4/80^+^ area in different groups. At least 10 randomly selected fields were assessed, and the results averaged for each kidney. ^*^ *p* < 0.05 versus sham, ^†^ *P* < 0.05 versus UIRI (n = 5). n–p) Inhibition of TLR4 reduces myofibroblast activation and renal fibrosis in vivo. Representative micrographs show renal expression and localization of α‐SMA by immunohistochemical staining (n) and collagens deposition by Masson's trichrome staining (o) in different groups as indicated. The areas between the dashed lines represent the corticomedullary junction of the kidney. Graphic presentation (p) shows the semi‐quantitative determination of renal α‐SMA^+^ area and fibrotic lesions in different groups. At least 10 randomly selected fields were assessed, and results averaged for each kidney. ^*^ *p* < 0.05 versus sham, ^†^ *P* < 0.05 versus UIRI (n = 5).

To further verify the role of TNC/TLR4/NF‐κB cascade in renal inflammation and fibrosis, we used TAK‐242 to block TLR4 signaling in the UIRI model (**Figure** **6**a). As shown in Figure 6b,c, serum creatinine and BUN were increased at 11 days after UIRI but decreased after inhibition of TLR4. TAK‐242 inhibited renal expressions of MR, Arg‐1, TNF‐α, and CCL2 after UIRI (Figure 6d,e). Furthermore, TAK‐242 repressed renal TLR4 expression and inhibited renal P65 phosphorylation and activation (Figure 6f,g). TAK‐242 also reduced TNC, fibronectin, and α‐SMA in the kidney after UIRI (Figure 6h,i). Notably, flow cytometry analysis showed a higher number of F4/80^+^/CD11b^+^/CD45^+^ macrophages in UIRI mice compared to the sham control group, while TAK‐242 treatment reduced the infiltration of renal macrophages (Figure 6j,k). Immunofluorescence staining for F4/80 showed that macrophage infiltration in the CMJ region was increased in UIRI, but reduced after TLR4 inhibition (Figure 6l,m). Immunostaining revealed that TAK‐242 repressed renal expression of α‐SMA (Figure 6n,p), which was mainly restricted to the CMJ region. TLR4 inhibition also mitigated collagen deposition and fibrotic lesions in the kidney (Figure 6o,p). These results indicate that TNC‐mediated TLR4 activation promotes renal inflammation and fibrosis in vivo.

**Figure 7:**
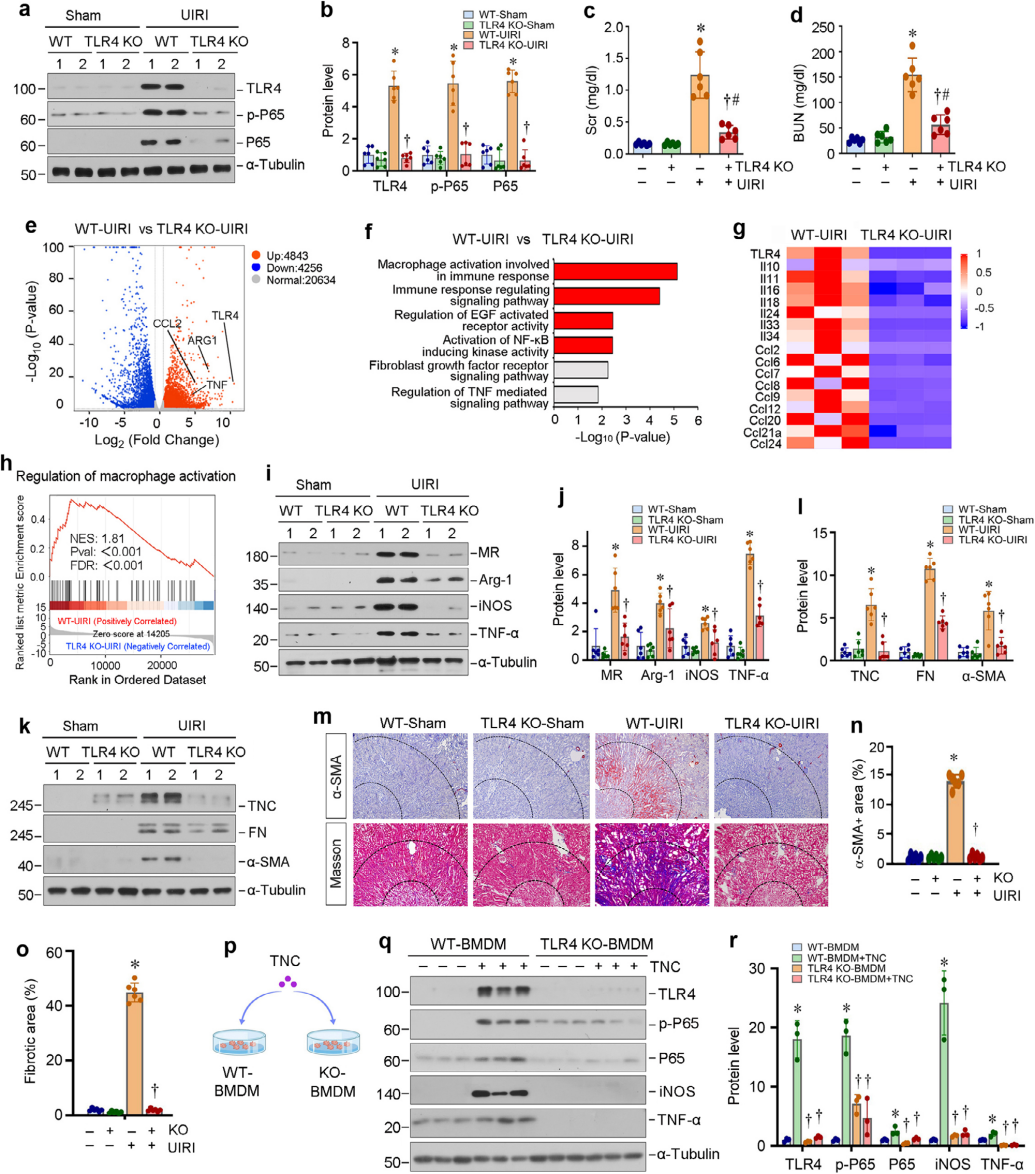
Knockout of TLR4 ameliorates renal inflammation and renal fibrosis in vivo. a) Western blot analyses show renal expression of TLR4, p‐P65, and P65 in different groups as indicated. Representative Western blot (a) and quantitative data b) are shown. ^*^ *p* < 0.05 versus WT‐Sham, ^†^ *P* < 0.05 versus WT‐UIRI (n = 6). c,d) Graphic presentations show serum creatinine (Scr) (c) and blood urea nitrogen (BUN) (d) levels in different groups as indicated. ^*^ *p* < 0.05 versus WT‐sham, ^†^ *P* < 0.05 versus WT‐UIRI, ^#^P < 0.05 versus TLR4 KO‐sham (n = 6). e) Volcano plot shows the differentially expressed genes of two groups (WT‐UIRI versus KO‐UIRI) as indicated by RNA‐seq. f) GO enrichment analysis reveals that several signaling pathways, as indicated, were enriched. g) The heat map shows differential gene expression of IL inflammatory cytokines and CCL chemokines in WT‐UIRI and TLR4 KO‐UIRI groups. h) GSEA enrichment analysis shows that the regulation of macrophage activation was enriched in the WT‐UIRI group. NES, normalized enrichment score; FDR, false discovery rate. i,j) Western blot analyses show renal expression of MR, Arg‐1, iNOS, and TNF‐α in different groups as indicated. Representative Western blot (i) and quantitative data (j) are shown. ^*^ *p* < 0.05 versus WT‐Sham, ^†^ *P* < 0.05 versus WT‐UIRI (n = 6). k,l) Western blot analyses show renal expression of TNC, FN, and α‐SMA in different groups as indicated. Representative Western blot (k) and quantitative data (l) are shown. ^*^ *p* < 0.05 versus WT‐sham, ^†^ *P* < 0.05 versus WT‐UIRI (n = 6). m) Representative micrographs show renal expression and localization of α‐SMA by immunohistochemical staining, and collagens deposition by Masson's trichrome staining in different groups as indicated. The areas between the dashed lines represent the corticomedullary junction of the kidney. n,o) Graphic presentation shows the semi‐quantitative determination of renal α‐SMA^+^ area (n) and fibrotic lesions (o) in different groups. At least 10 randomly selected fields were assessed, and the results were averaged for each kidney. ^*^ *p* < 0.05 versus WT‐Sham, ^†^ *P* < 0.05 versus WT‐UIRI (n = 6). p) The Diagram shows the experimental protocol. BMDMs were isolated from WT and TLR4‐KO mice, and then stimulated with TNC (100 ng ml^−1^) for 48 h in vitro. q,r) Western blot analyses show renal expression of TLR4, p‐P65, P65, iNOS and TNF‐α in different groups as indicated. Representative Western blot (q) and quantitative data (r) are shown. ^*^ *p* < 0.05 versus WT‐BMDM, ^†^ *P* < 0.05 versus WT‐BMDM + TNC (n = 3).

We further investigated the role of TNC/TLR4 in renal inflammation and fibrosis by using TLR4 knock‐out (KO) mice. Western blotting confirmed that TLR4‐KO inhibited NF‐κB signaling, as protein expressions of TLR4, p‐P65, and P65 were inhibited after UIRI (**Figure** **7**a,b). Mice with TLR4‐KO exhibited improved serum creatinine and BUN levels at 11 days after UIRI (Figure 7c,d). By using an RNA‐seq transcriptome profiling approach, we found substantial differences in gene expression between wild‐type (WT) and TLR4‐KO mice after UIRI (Figure 7e). Compared with the WT‐UIRI group, the mRNA levels of TLR4, CCL2, Arg‐1, and TNF‐α were downregulated in the TLR4 KO‐UIRI group (Figure 7e). Gene ontology (GO) analysis revealed that several inflammation‐related signaling pathways were enriched in WT‐UIRI mice, including macrophage activation involved in immune response, immune response regulating signaling pathway, regulation of EGF‐activated receptor activity, and activation of NF‐κB inducing kinase activity (Figure 7f). In addition, the gene heatmap further showed that compared with the WT‐UIRI group, the gene expressions of various IL cytokines and CCL chemokines were downregulated (Figure 7g). GSEA revealed the regulation of macrophage activation was enriched in the WT‐UIRI group (Figure 7h). Western blotting indicated that TLR4‐KO effectively inhibited the expression of several major inflammation‐related proteins, such as MR, Arg‐1, iNOS, and TNF‐α (Figure 7i,j). In addition, TLR4‐KO alleviated the expression of fibrosis‐related proteins such as TNC, FN, and α‐SMA after UIRI (Figure 7k,l). Immunostaining exhibited that TLR4‐KO inhibited renal expression of F4/80, CCR2, α‐SMA, and reduced collagen deposition in fibrotic kidneys (Figure S7a,b,m–o). These results show that TNC‐mediated TLR4 activation is required for renal inflammation and fibrosis after injury.

To further investigate the role of TNC/TLR4 in mediating macrophage activation, we isolated and cultured BMDMs from WT and TLR4‐KO mice and stimulated with TNC in vitro (Figure 7p). As shown in Figure 7q,r, TNC activated TLR4/NF‐κB signaling and upregulated the expressions of iNOS and TNF‐α in WT BMDMs. However, in the BMDMs derived from TLR4 KO mice, TLR4/NF‐κB activation and induction of iNOS and TNF‐α were abolished even after TNC stimulation (Figure 7q,r). These results indicate that TNC‐mediated macrophage activation is dependent on TLR4 signaling.

### 2.8 TLR4 Activation in Macrophages is Essential for Renal Inflammation and Fibrosis

**Figure 8:**
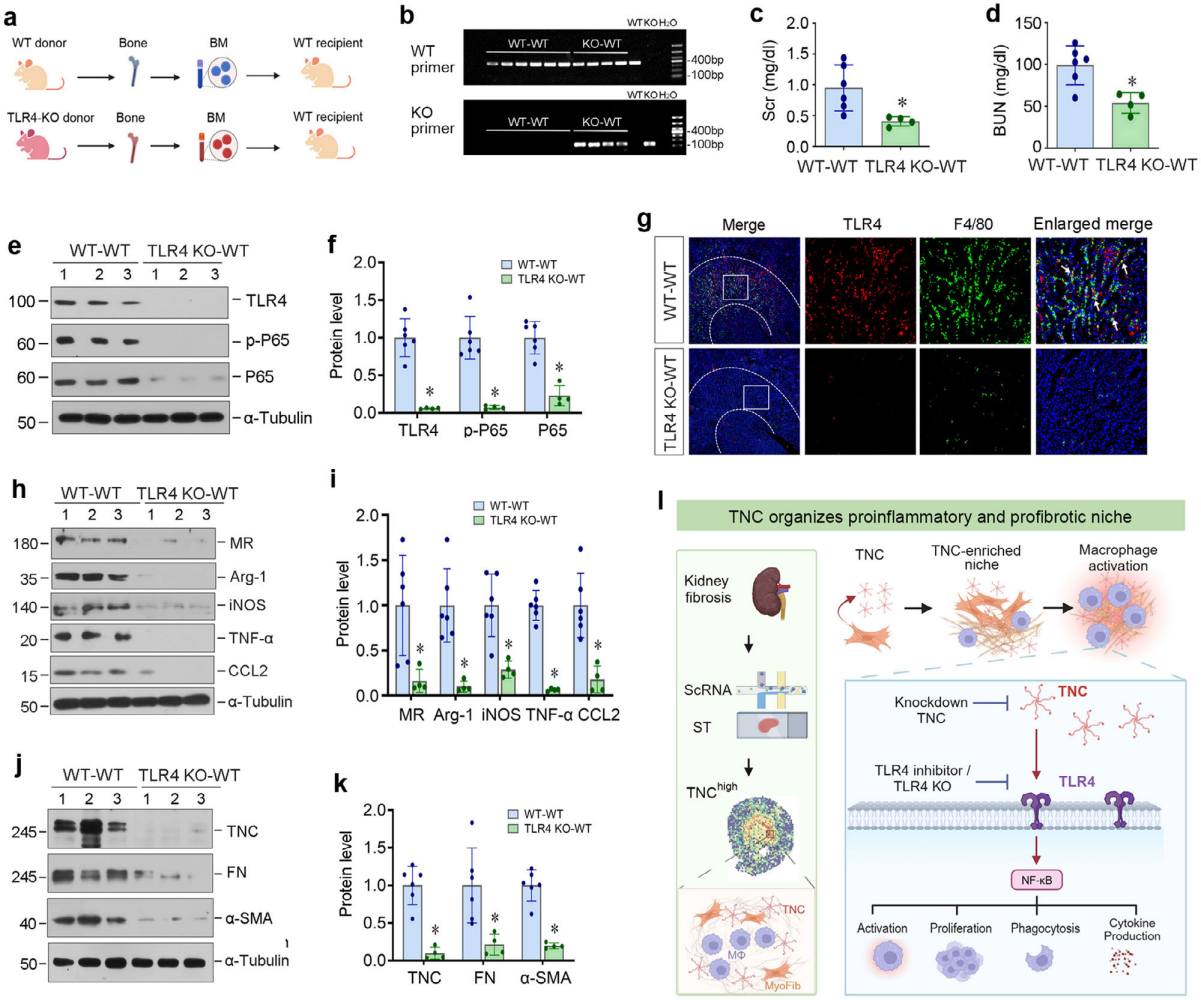
TLR4 knockout in macrophages attenuates renal inflammation and renal fibrosis in vivo. a) The diagram shows the experimental protocol. Bone marrow chimera models were established by transplanting the WT bone marrow to WT mice, or TLR4 KO bone marrow to WT mice. Mice were irradiated at a single dose of 1100 Rads and then underwent bone marrow transplantation. After 8 weeks of successful transplantation, a unilateral ischemia‐reperfusion (UIRI) model was established. b) PCR‐based identification of kidney genotypes in the recipient mice of bone marrow transplantation models using TLR4 mutation site primers and wild‐type site primers, respectively. c,d) Graphic presentations show serum creatinine (Scr) (c) and blood urea nitrogen (BUN) (d) levels in different groups as indicated at 11 days after IRI. ^*^ *p* < 0.05 versus WT‐WT (n = 4–6). e,f) Western blot analyses show renal expression of TLR4, p‐P65, and P65 in different groups as indicated. Representative Western blot (e) and quantitative data (f) are shown. ^*^ *p* < 0.05 versus WT‐WT (n = 4–6). g) Representative micrographs show renal expression and co‐localization of TLR4 and F4/80 by immunofluorescence staining in different groups as indicated. The areas between the dashed lines represent the corticomedullary junction of the kidney. h,i) Western blot analyses show renal expression of MR, Arg‐1, iNOS, TNF‐α, and CCL2 in different groups as indicated. Representative Western blot (h) and quantitative data (i) are shown. ^*^ *p* < 0.05 versus WT‐WT (n = 4–6). j,k) Western blot analyses show renal expression of TNC, FN, and α‐SMA in different groups as indicated. Representative Western blot (j) and quantitative data (k) are shown. ^*^ *p* < 0.05 versus WT‐WT (n = 4–6). l) A schematic diagram shows a crucial role of TNC in organizing the proinflammatory and profibrotic niche. By integrating single‐cell RNA sequencing and spatial transcriptomics, we unveil TNC as a central organizer of the proinflammatory and profibrotic niche in kidney fibrosis. TNC promotes macrophage activation through TLR4/NF‐κB signaling, leading to macrophage activation, proliferation, and cytokine production.

As the TLR4 KO used was a global knockout model, we further clarified the role of the TNC‐triggered TLR4 activation in macrophages in renal inflammation and fibrosis. To this end, we established a bone marrow chimera model by transplanting the bone marrow from either WT or TLR4 KO to WT mice (**Figure** **8**a). The WT recipient mice were subjected to X‐ray irradiation to ablate bone marrow hematopoietic system, which was reconstructed by WT‐ and TLR4‐KO‐derived bone marrow, forming a new chimera. Genetic analysis of bone marrow chimeras (Figure 8b) confirmed successful hematopoietic engraftment, as evidenced by the co‐detection of both donor‐ and recipient‐specific genotypes in kidney tissue. We found that compared to the WT mice transplanted with WT bone marrow (WT‐WT) group, the WT recipient mice transplanted with TLR4 KO bone marrow (TLR4 KO‐WT) group showed a significant decrease in blood creatinine and BUN levels after UIRI (Figure 8c,d). Western blot analyses showed that TLR4/NF‐κB activation in TLR4 KO‐WT mice was attenuated compared to WT‐WT mice (Figure 8e,f). Immunofluorescence co‐staining for TLR4 and F4/80 confirmed a decrease in infiltrating macrophages in the renal interstitium of the TLR4 KO‐WT mice (Figure 8g). In addition, renal expression of MR, Arg‐1, iNOS, TNF‐α, and CCL2 was decreased in the TLR4 KO‐WT group as well (Figure 8h,i). Similarly, the expressions of TNC, FN, and α‐SMA were inhibited in the TLR4 KO‐WT group (Figure 8j,k). Taken together, TLR4 activation in macrophages is primarily responsible for mediating renal inflammation and kidney fibrosis after injury.

## 3 Discussion

Kidney fibrosis often initiates at distinct focal sites by forming the fibrotic niche, in which inflammatory cells and fibroblasts are activated and markedly expanded. In this study, we utilized an integrated scRNA‐seq and ST approach to dissect the cellular heterogeneity, spatial organization, and molecular interactions in the fibrotic kidney. By building the transcriptomic atlas of normal and fibrotic kidneys, we identified and characterized a predominant proinflammatory and profibrotic microenvironment where the fibroblasts and macrophages are at close proximity in the corticomedullary junction of the fibrotic kidneys after ischemic injury (Figure 2). Furthermore, we demonstrated that the TNC secreted by fibroblasts acts as the primary organizer of the proinflammatory and profibrotic niche, activating the macrophages through triggering TLR4/NF‐κB signaling (Figure 8l). Our findings for the first time highlight the complex interplay among fibroblasts, extracellular microenvironment, and macrophages within the fibrogenic niche that drives kidney inflammation and fibrosis. These studies provide novel insights into understanding the cellular makeup, spatial organization, intercellular communication and molecular interactions within the fibrogenic niche.

Kidney fibrosis is typically preceded by the infiltration of various inflammatory cells.^[^ ^2^, ^4^, ^34^ ^]^ It is well documented that the severity and stages of CKD are closely related to the degree of inflammatory cell infiltration, particularly the macrophages. Notably, the activation status and function of macrophages are clearly influenced by their surroundings, in which they interact with other cell types and components of the so‐called immune microenvironment.^[^ ^35^, ^36^ ^]^ In this study, by integrating scRNA‐seq and high‐resolution ST data, we have illustrated an intricate and spatial localization of the macrophages and fibroblasts in the CMJ of the fibrotic kidney, a region that suffered from the most severe damage in this IRI model (Figure 2).^[^ ^20^ ^]^ These results illustrate the inflammatory and profibrotic cellular landscapes in the kidney after injury, and suggest that the fibrogenic niche not only is a safe haven for fibroblast expansion but also an immune milieu for macrophage activation. Consistently, recent studies also demonstrate the emergence of the fibrotic microenvironment with unique transcriptional signatures in injured proximal tubules and fibroblast‐macrophage foci after kidney injury.^[^ ^37^, ^38^ ^]^ Collectively, these findings underscore that renal inflammation and fibrosis are indivisible in CKD, and they are essentially coupled by sharing the same setting.

The present study identifies TNC as the primary organizer of the proinflammatory and profibrotic microenvironment, because it not only promotes fibroblast activation and tubular cell dedifferentiation as previously reported,^[^ ^18^, ^19^ ^]^ but also induces macrophage activation, proliferation, migration, phagocytosis, and cytokine production (Figure 3). This notion is supported by several lines of evidence. First, TNC is highly upregulated in a diverse array of CKD, and it localizes focally at specific sites within the kidney where injury occurs, consistent with the notion of the fibrotic niche.^[^ ^18^ ^]^ Furthermore, by using dc‐KTS from the control and fibrotic kidneys, we found that TNC‐enriched extracellular microenvironment promotes macrophage activation and proliferation, whereas TNC‐depleted dc‐KTS hampers the activation of macrophages and the expression of inflammatory cytokines (Figure 3). Moreover, exogenous TNC protein directly induces macrophage activation, proliferation, migration, and enhances their phagocytic function (Figure 4), while other ECM components, such as collagen I and fibronectin, exhibit no effect on macrophage activation under the same conditions (Figure S5, Supporting Information). In addition, TNC induces and activates TLR4, one of the evolutionarily conserved receptor family that not only plays a role in regulating innate and adaptive immune responses, but also is pivotal in the pathogenesis of non‐infectious inflammatory diseases of various organs such as the cardiovascular system, kidneys, lungs, and liver.^[^ ^39^, ^40^, ^41^ ^]^ Collectively, these studies suggest that TNC, acting as a damage‐associated molecular pattern (DAMP), binds to and activates TLR4 in macrophages and induces their activation. Therefore, TNC organizes special focal sites or areas where both fibroblasts and macrophages are activated and expanded. In these focal areas, inflammation and fibrosis are reciprocally stimulated, leading to the formation of the fibrotic foci in CKD.^[^ ^7^ ^]^

It should be stressed that TNC also promotes fibroblast activation and proliferation via integrin/FAK/MAPK signaling.^[^ ^18^ ^]^ In this context, it is reasonable to speculate that after kidney injury, TNC organizes a proinflammatory and profibrotic nice, in which it promotes both macrophage and fibroblast activation simultaneously and in parallelly, but not necessarily sequentially. The present study provides several key advances beyond existing literature. First, by integrating scRNA‐seq and ST, particularly via Visium HD technology, we identify the TNC‐enriched niche with precision as the key location where fibroblast‐macrophage cross‐talk is operated through direct spatial proximity, a dimension previously unexplored by traditional techniques. Second, we establish that TNC acts as a DAMP to activate macrophages via TLR4/NF‐κB signaling (Figures 3 and 4) and as a profibrotic factor to activate fibroblasts,^[^ ^18^ ^]^ revealing its role in coordinating both fibrogenic and inflammatory responses within the same location. Third, in contrast to the previous fibroblast‐centric view, we demonstrate with single‐cell resolution that TNC unites renal inflammation and fibrosis by orchestrating proinflammatory and profibrotic niche formation. These findings collectively redefine TNC as a master regulator bridging spatial organization with inflammatory‐fibrotic coupling in the evolution of CKD.

This study also addresses the mechanism underlying macrophage activation induced by the TNC‐enriched microenvironment. As TNC activates the TLR4 on the surface of macrophages, leading to NF‐κB signaling activation, we blocked TNC actions by using TAK‐242, a small molecule TLR4 antagonist. TAK‐242 selectively disrupts TLR4 signaling by directly binding to its intracellular Toll/interleukin‐1 receptor (TIR) domain, resulting in impaired recruitment of TIR domain‐containing adaptor protein (TIRAP) and inactivation of NF‐κB signaling,^[^ ^42^ ^]^ leading to the inhibition of TNF‐α, IL‐6, and iNOS expression. Indeed, TAK‐242 effectively abolishes macrophage activation in vitro (Figure 4) and inhibits kidney inflammation and fibrosis after injury in vivo (Figure 6). Furthermore, pharmacological blockade of TLR4 using a neutralizing antibody completely abolished TNC‐induced macrophage proliferation and activation (Figure S6, Supporting Information). Consistently, either knockdown of TNC or knockout of TLR4 in vivo blocks the activation of TLR4/NF‐κB signaling and represses renal inflammation, alleviates renal fibrosis, and preserves renal function (Figures 5, 6, 7). Furthermore, to eliminate the possibility that TLR4 signaling in other cell types, such as tubular cells, may also participate in regulating kidney inflammation and fibrosis, we adopted a bone marrow transplantation protocol and constructed chimeric mice with TLR4‐/‐ bone marrow. We found that compared to WT‐WT controls, mice transplanted with TLR4‐/‐ bone marrow are protected against kidney inflammation and fibrosis (Figure 8). Together with the observations that TLR4 is primarily induced in F4/80^+^ macrophages in fibrotic kidneys (Figure 5), these results indicate that TNC‐enriched microenvironment specifically activates macrophages by triggering TLR4/NF‐κB signaling.

This present study may have some potential limitations. For example, as numerous types of cells are involved in regulating kidney inflammatory responses after injury, the immune cells derived from bone marrow would contain many types of cells besides macrophages, thereby potentially complicating data interpretation. Additionally, our bulk analyses using flow cytometry cannot distinguish whether inhibition of inflammation by TNC knockdown, TAK‐242, and TLR4 knockout is due to the collapse of specific proinflammatory macrophage subsets, their transdifferentiation into different phenotypes, or being replaced, these questions require future studies at the single‐cell level by scRNA‐seq. However, as TLR4 is primarily induced in F4/80^+^ macrophages in the fibrotic kidney (Figure 5), it is unlikely that TLR4 signaling in other types of cells plays a major role in mediating TNC‐triggered inflammation, although we cannot completely exclude that possibility. Furthermore, as TLR4 has several ligands, we cannot rule out that other non‐TNC ligands of TLR4 may also play a role in mediating macrophage activation in vivo, despite that knockdown of TNC completely inhibited BMDM activation in an ex vivo system (Figure 3). Additionally, while donor‐derived cell engraftment in kidneys was confirmed, the extent of peripheral reconstitution was not quantitatively assessed. Another issue is that macrophages may not only promote kidney injury by inciting inflammation, but also directly cause fibrotic lesions by promoting macrophage‐myofibroblast transformation.^[^ ^43^ ^]^ It is conceivable to speculate that TNC, as the organizer of the fibrotic niche, promotes CKD progression by affecting multiple types of cells, such as fibroblasts, macrophages and tubular cells in a spatially defined fashion.

In summary, using an integrated scRNA‐seq and ST profiling approach, we identified and defined a TNC‐enriched, proinflammatory, and profibrotic microenvironment that plays a crucial role in initiating renal inflammation and fibrosis after kidney injury. We show that fibroblast‐derived TNC acts as the primary organizer that orchestrates the formation of the proinflammatory and profibrotic niche, and triggers macrophage activation via TLR4/NF‐κB signaling. These findings suggest that targeting TNC/TLR4 axis could be a new strategy to disrupt the fibrotic processes and pave novel way for developing effective therapeutic interventions in combating against fibrotic CKD.

## 4 Experimental Section

### Animal Models

Mice with a deficiency of TLR4 (*Tlr4^−/−^*) in C57BL/10ScNJ background were purchased from GemPharmatech (Nanjing, China). The other animals were obtained from the Southern Medical University Animal Center in Guangzhou, China. Male C57BL/6 mice underwent unilateral ischemia‐reperfusion injury (UIRI), and at day 10 post‐UIRI, the contralateral intact kidney was removed.^[^ ^44^ ^]^ The mice were euthanized 11 days after UIRI, and serum and kidney tissues were collected for various analyses. For inhibition of TLR4 signaling, mice were daily injected intraperitoneally with TAK‐242 at 8 mg kg^−1^ body weight from day 4 after UIRI, and sacrificed at 11 days post‐UIRI. Bone marrow chimera models were established by transplanting the wild‐type (WT) bone marrow to WT mice or TLR4 KO bone marrow to WT mice. Mice were irradiated at a single dose of 1100 Rads and then underwent bone marrow transplantation. After 8 weeks of successful transplantation, the UIRI model was established.^[^ ^45^, ^46^ ^]^ Animals were randomly assigned to different treatment groups. All animal experiments were conducted with approved protocols (#NFYY‐2021‐0340 and HTSW210317) by the Experimental Animal Committee at Nanfang Hospital, Southern Medical University.

### Preparation of the Kidney Tissue Scaffold

The decellularized kidney tissue scaffold (dc‐KTS) was prepared according to the well‐established protocol reported previously.^[^ ^47^ ^]^ Mice were injected intravenously with either control‐shRNA or TNC‐shRNA plasmid vectors at 4 days after UIRI. Kidneys from different groups were arterially perfused in situ using phosphate‐buffered saline (PBS) to remove the blood. The kidneys were then sliced to a uniform thickness. These kidney slices underwent a series of decellularization procedures using deoxyursocholic acid and Triton X‐100.^[^ ^18^ ^]^ Macrophages were subsequently seeded onto the dc‐KTS and incubated for 2 days. Cell lysates were then prepared and analyzed by Western blot analysis.

### Cell Culture and Treatment

The culture of bone marrow‐derived macrophages (BMDMs) was conducted as previously described.^[^ ^48^ ^]^ In brief, femurs from adult mice aged 6–8 weeks were dissected to extract the bone marrow, which was subsequently cultured in Dulbecco's modified Eagle's medium (DMEM) supplemented with 20% fetal bovine serum and 30% L929 conditional medium to induce BMDM differentiation. The purity of BMDMs was assessed by staining with CD11b and F4/80 antibodies. Macrophages were incubated with TNC at different dosages for various periods of time as indicated. For some experiments, macrophages were pretreated with TAK‐242 (5 nM), followed by incubating with vehicle or TNC (R&D, Catalog#: 3358‐TC, 100 ng ml^−1^, with a purity > 95% and endotoxin level <0.10 EU per 1 µg of the protein). For some experiments, BMDMs were pretreated with TLR4‐blocking antibody (Invitrogen, Catalog#: 14‐9924‐82, 5 µg ml^−1^) or isotype IgG (Isotype) for 2 h, then treated with TNC (100 ng ml^−1^) for 48 h. For some experiments, macrophages were incubated with vehicle or collagen I (Abclonal, Catalog#: RP01842) or fibronectin (R&D, Catalog#: 1918‐FN‐02 M) for 48 h.

### Co‐Immunoprecipitation Assay

BMDMs were homogenized in 500 µl NP40 lysis buffer supplemented with protease inhibitors and kept on ice for 30 min. Cell lysates were collected using a sterile cell scraper and clarified by centrifugation (12000 × g, 10 min, 4 °C). Equal protein aliquots of the supernatant were pre‐cleared and incubated with target‐specific antibodies for 60 min at 4 °C with gentle rotation. Protein A/G PLUS‐Agarose immunoprecipitation beads (sc‐2003, Santa Cruz Biotechnology) were then added to capture antigen‐antibody complexes overnight at 4 °C under constant agitation. After three stringent washes with PBS, the immunoprecipitated proteins were eluted and analyzed by immunoblotting.

### Bulk RNA Sequencing and Data Analyses

Total RNA of the kidneys or cells was extracted with Trizol (Invitrogen). The RNA integrity number (RIN) was determined by an Agilent Bioanalyzer 4150 system (Agilent Technologies, CA). Paired‐end libraries were prepared using an ABclonal mRNA‐seq Lib Prep Kit (ABclonal, China) according to the manufacturer's instructions. RNA‐seq was carried out on Novaseq 6000 (illumina) and MGISEQ‐T7 (BGI), and 150 bp paired‐end reads were generated. Each RNA‐seq read was mapped to the mouse genome using HISAT2 software (http://daehwankimlab.github.io/hisat2/).^[^ ^49^ ^]^ FeatureCounts (http://subread.sourceforge.net/) was used to count the reads numbers mapped to each gene. The FPKM (fragments per kilobase per million mapped reads) of each gene was then calculated based on the length of the gene and the reads count mapped to this gene. Differential expression analysis was performed using the DESeq2 (http://bioconductor.org/packages/release/bioc/html/DESeq2.html), and adjusted *P* < 0.05 was considered as a significantly differential expression.

The R software package cluster Pro filer was used for the gene ontology (GO) and Kyoto Encyclopedia of genes and genomes (KEGG) enrichment analysis.^[^ ^50^ ^]^ Enrichment analyses were applied based on the Fisher’ exact test, when *P* < 0.05, it was considered that the GO and KEGG functions were significantly enriched. Gene set enrichment analysis (GSEA) was performed by GSEA software with default parameters against KEGG gene sets.

### Single Cell RNA Sequencing and Data Processing

Fresh kidney tissue was diced into 1 mm^3^ pieces and immersed in a collagenase buffer at 37 °C for 30 min. The reaction was halted with equal parts cold PBS/10% FBS. After viability assessment, the 10x Genomics platform was employed for transcriptomic sequencing. The procedure involved three primary steps: 1) cDNA amplification; 2) library construction; 3) sequencing on the Illumina NovaSeq system.

The CellRanger's pipelines (version 7.1.0 https://www.10xgenomics.com), tailoring procedures were utilized for Chromium Single Cell Gene Expression data, in which sequence alignment, UMI counting, and cell barcode identification were integral, ensuring an accurate interpretation and further analysis of single‐cell data. Single‐cell data analysis was carried out utilizing Seurat (version 4.4.0).^[^ ^51^ ^]^ Soupx (version 1.6.2) was employed to eliminate environmental RNA contamination and calculate cell expression profiles post background correction, utilizing standard parameters.^[^ ^52^ ^]^ DoubletFinder (Version 2.0.3) was put to use to identify and expel heterotypic doublet peaks.^[^ ^53^ ^]^

Normalization and scaling of gene expression were executed using the NormalizeData and ScaleData functions, respectively. The foremost 2000 differential genes were chosen using FindVariableFeatures for PCA analysis. Dimension reduction was performed on the initial 20 principal components using RunPCA. Batch effects were mitigated with the R package harmony(version 1.1.0),^[^ ^54^ ^]^ and clustering was conducted with the FindClusters function at a resolution of 0.8.

The following downstream analyses were then carried out. 1) Differential gene analysis. The FindAllMarkers function was utilized in Seurat to discover differentially expressed genes (DEGs) across all clusters.^[^ ^51^ ^]^ 2) Enrichment analysis. The clusterProfiler^[^ ^50^ ^]^ (version 4.8.3) R package was employed to conduct KEGG pathway enrichment analysis on the DEGs, with a significance threshold set at a p‐value of 0.05. Gene Set Variation Analysis (GSVA) was conducted utilizing the GSVA package (version 1.48.3). 3) Cell‐cell interaction analysis. Intercellular communication within the dataset was illuminated through the application of CellChat,^[^ ^26^ ^]^ which operates based on its intrinsic database of ligand‐receptor pairs, encompassing three major categories of intercellular communication: cell‐cell contact, ECM‐receptor, and secreted signaling. This database was enriched with Tnc‐associated pairs from literature.^[^ ^17^, ^27^, ^28^, ^29^ ^]^ 5) Cell type correlation analysis. To validate cell type annotations, cross‐dataset correlation analysis was performed with a published IRI scRNA‐seq dataset.^[^ ^21^ ^]^ Shared genes were identified, and average expression levels per cell type were used to compute Pearson correlation coefficients, assessing transcriptional similarity between matched cell types.

Additionally, the analysis was supplemented with UUO samples from studies by other groups,^[^ ^22^, ^55^, ^56^, ^57^, ^58^, ^59^, ^60^ ^]^ and IRI samples from Aggarwal et al.,^[^ ^21^ ^]^ with detailed sample information provided in Supplementary Table S1. For UUO samples, cells with 400–6000 expressed genes was retained, fewer than 15000 UMIs, <10% hemoglobin genes, <30% mitochondrial genes, and gene complexity (defined as log10 (nFeature_RNA) / log10 (nCount_RNA) >0.8; for IRI samples, cells with 400–7000 expressed genes, <30% mitochondrial genes, <30% ribosomal genes, and gene complexity >0.8 were retained.

### Spatial Transcriptomics Visium Data Processing

Mouse kidney samples stored in a frozen state were embedded with OCT compound and stored at −80 °C. For the preparation of sections for Visium Spatial Transcriptomics sequencing, samples were equilibrated at −18 °C and a 10 µm thick section was cut onto the active sequencing area (6 mm x 6 mm) of a spatial barcoded slide. Furthermore, H&E staining and photographic imaging were performed on the frozen slices to retain histological information of the sample.

Primers with spatial barcodes were used to capture mRNA released from cleared tissue. The captured mRNA reacted with the primers to synthesize the corresponding cDNA, which was then amplified by PCR. Upon successful verification, they underwent library construction. Sequencing was carried out using the Illumina NovaSeq S4 sequencing platform. Dual index sequencing was performed in accordance with the guidelines of 10x Genomics Visium manufacturer (PN‐1000185, lot number 155 614, revision D), with the goal of obtaining 125 million reads. The raw data (FASTQ format) obtained from sequencing were aligned with the mm10 reference genome using appropriate alignment software (such as Bowtie, STAR, etc.), manually aligned with the corresponding H&E images, and spatial information was associated with gene expression data. Normalization was performed using the 10x Genomics Space Ranger count software (Spatial 3′ v1; spaceranger‐1.2.1).

The raw sequencing data (FASTQ files) and H&E images of the tissue sections were input into the Space Ranger software. A comparison of raw data with the reference genome was performed, and tissue detection was carried out to obtain tissue structure information. Baseline calibration and barcode/UMI statistics were applied to gene expression data for sequencing depth correction and experimental quality assessment.

The following analysis was next carried out. 1) Data preprocessing. ST data preprocessing was performed using the R package Seurat and Semla.^[^ ^51^, ^61^ ^]^ 2) Enrichment analysis. An in‐depth enrichment analysis was performed by maintaining consistency with the scRNA‐seq method section described previously. 3) Spatial transcriptomics deconvolution. The deconvolution was performed using the Spacexr package (version 2.2.1), based on the Robust Cell Type Decomposition (RCTD).^[^ ^25^ ^]^ 4) Calculation of gene expression correlation. The Pearson correlation coefficient was utilized to measure the correlation between genes at spatial locations. The top 10 molecules were then selected with the highest correlation to the target gene for further analysis. To ensure the statistical significance of the results, a threshold was set for the *P*‐value at 0.01. 5) NMF analysis. Non‐negative matrix factorization (NMF) was performed on integrated spatial transcriptomic data using the RunNMF function (Singlet R package, v0.99.8). The optimal number of factors was determined with the RankPlot function.

For the UUO spatial transcriptomics data derived from the study by Yasui et al.,^[^ ^57^ ^]^ the same preprocessing workflow was applied. The only differences were the use of the top 20 principal components for RunPCA and a clustering resolution of 0.8.

### Visium HD Data Processing

Formalin‐fixed paraffin‐embedded (FFPE) kidney tissue blocks were obtained from C57BL/6 male mice (8 weeks old) subjected to UIRI for 10 days, consistent with the animal model used in the preceding scRNA‐seq and 10x Visium experiments. Serial 5 µm sections were prepared from the FFPE blocks using a Leica RM2255 microtome, following the Visium HD Spatial Gene Expression FFPE Tissue Preparation Guide (10x Genomics, CG000684 Rev A), and mounted onto Sigma‐Aldrich Poly Prep Slides. The slides were air‐dried overnight at room temperature and then incubated at 60 °C for 2 h. Subsequently, tissue sections underwent deparaffinization, hematoxylin and eosin (H&E) staining, and brightfield imaging using a Leica Aperio Versa 8 microscope, strictly adhering to the 10x Genomics Visium HD Spatial Gene Expression FFPE protocol, in order to preserve histomorphological features for spatial transcriptomic analysis.

The mouse whole transcriptome probe panel was applied to the tissue sections. After hybridization of probes to target mRNA sequences and subsequent ligation, the slides underwent RNase treatment and tissue permeabilization using the Visium HD instrument. The ligated probe complexes were then captured by spatially barcoded oligonucleotides embedded within the active capture areas of the slide, enabling the generation of high‐resolution spatial transcriptomic libraries. These libraries were subsequently sequenced on an Illumina platform according to the manufacturer's protocol.

After cDNA library construction and high‐throughput sequencing, raw sequencing data underwent initial quality control using in‐house scripts, which included assessment of overall data quality and evaluation of GC content across sequencing cycles. Subsequently, FASTQ files and histological images were analyzed using the FFPE “count” pipeline in 10x Genomics Space Ranger software (v3.1), where sequencing reads were aligned to the mouse reference genome (mm10) using a short‐read probe alignment algorithm.

The following analysis was next carried out. 1) Data preprocessing. We performed integrated analysis of Visium HD spatial transcriptomic data (control and UIRI10D kidney samples) using Seurat and Semla. Data were normalized (NormalizeData), scaled (ScaleData), and subjected to variable feature selection (FindVariableFeatures) before PCA and CCA‐based integration (IntegrateLayers). Clustering used the top 30 PCs (resolution = 0.8). 2) Spatial deconvolution & NMF. Spatial deconvolution was performed using the standard pipeline (with doublet_mode = “doublet” for HD spot size). NMF analysis used Singlet (v0.99.8 (RunNMF/RankPlot)), matching the standard Visium workflow.

### Knockdown of TNC In Vivo

The knockdown of TNC expression in vivo was conducted by using an shRNA‐mediated approach, as previously reported.^[^ ^19^ ^]^ Male BALB/c mice were divided into three groups, each consisting of six animals: 1) mice subjected to a sham operation, 2) UIRI mice administered with control shRNA, and 3) UIRI mice administered with TNC‐shRNA. Mice were subjected to tail‐vein injections of either pLVX‐shTNC or control (pLVX‐control) plasmids at 4 days after UIRI.

### EdU Incorporation Assay

The 5‐ethynyl‐2′‐deoxyuridine (EdU) incorporation assay was conducted according to a routine protocol. Briefly, cells were seeded onto six‐well plates and treated with TNC as indicated. Subsequently, the cells were incubated with EdU (10 µM) for 6 h, and then fixed. They were then incubated with the EdU reaction mixture for 30 min at room temperature. To visualize the stained samples, Hoechst reaction solution was added to each well. The stained samples were observed using an Eclipse E600 epifluorescence microscope equipped with a digital camera (Nikon, Tokyo, Japan).

### Flow Cytometry Analysis

Macrophages were incubated with TNC for 48 h. Subsequently, they were rinsed with cold PBS and stained using the Cycletest Plus DNA Reagent Kit (Becton Dickinson, CA, USA) according to the manufacturer's guidelines. The cell cycle distribution was assessed using a FACSCanto II Flow cytometry (Becton Dickinson, CA), and the data were analyzed by using the ModFit LT3.3 software. A total of 10000 events per sample were recorded for each experimental trial. For analyzing mouse kidney macrophages, single‐cell suspensions from the kidneys were made according to an established protocol.^[^ ^62^ ^]^ After washing with cold PBS, cells were stained with fluorescent‐conjugated antibodies (F4/80‐PE, CD11b‐FITC, CD45‐APC). Acquisition was performed on a FACSCanto II Flow cytometry (Becton Dickinson, CA, USA). Analysis was performed using the FlowJo software.

### Macrophage Phagocytosis Experiment

Phagocytosis was detected through flow cytometry as previously reported.^[^ ^63^ ^]^ Macrophages were incubated with TNC for 48 h, and fluorescent microspheres labeled with green fluorescent protein (GFP) were then added. After incubation for 1 h, cells were collected for flow cytometry detection. Analysis was performed using the FlowJo software.

### Transwell Migration Assay

The macrophage migration assay was performed using 24‐well Transwell chambers (Corning). Macrophages were incubated with TNC and cultured in the upper chamber for 48 h. The migratory capacity was assessed by quantifying the number of cells that traversed the membrane after staining with hematoxylin. Five fields were randomly chosen and examined under an inverted microscope. The results were averaged for each sample, and the experiment was replicated three times.

### Western Blot Analyses

Protein expression was analyzed by Western blot analysis as described previously.^[^ ^64^, ^65^ ^]^ The antibodies used are listed in Supplementary Table S2.

### Quantitative Real‐Time RT‐PCR

Total RNA was isolated, and qPCR analysis was performed using an ABI PRISM 7000 Sequence Detection System, as previously described.^[^ ^47^ ^]^ The mRNA levels of various genes were determined by normalizing with β‐actin. The sequences of primer pairs are listed in Supplementary Table S3.

### Histology, Immunohistochemical, and Immunofluorescence Staining

Paraffin‐embedded sections of mouse kidneys were prepared using a standard procedure. Masson's trichrome staining (MTS) reagents were employed to stain the sections. Immunohistochemical and immunofluorescence staining were conducted by following previously established protocols. Antibodies used are listed in Supplementary Table S2.

### Statistical Analyses

All data examined were expressed as mean ± SEM. Statistical analyses of the data were performed using SPSS Statistics (SPSS Inc, Chicago, IL). Group comparisons were made through *t* test, or by employing one‐way ANOVA followed by Fisher's least significant difference test or Dunnett T3 test. *P* < 0.05 was considered significant.

## Conflict of Interest

The authors declare no competing interests.

## Author Contributions

L.L., J.L., and Y.Z. contributed equally to this work. Y.L. conceived the study. H.F., L.L., and Y.L. designed the study. L.L., J.L.L., Z.Y., J.H., K.W., L.L., Y.P., H.Z, X.H., L.Z., and X.L. carried out experiments. Y.Z. analyzed the single‐cell and spatial transcriptomics data, and L.L. analyzed the bulk transcriptomics data. L.L., J.L., H.F., and F.F.H. analyzed experimental data. L.L., J.L., and Y.Z. made the figures. L.L., Y.Z., and Y.L. drafted and revised the paper; all authors approved the final version of the paper.

## Code Availability

The custom code and quality control metrics used in this study are publicly available in the GitHub repository at: https://github.com/YuxiZhang‐0113/Tnc_Fibrogenic_Niche‐YuxiZhang.

## Supporting information



Supporting Information

## Data Availability

The raw transcriptomic data, including bulk RNA‐seq, single‐cell RNA‐seq, and spatial transcriptomics data, reported in this paper have been deposited in the Genome Sequence Archive (Genomics, Proteomics & Bioinformatics 2021) in National Genomics Data Center (Nucleic Acids Res 2022), China National Center for Bioinformation / Beijing Institute of Genomics, Chinese Academy of Sciences (GSA: CRA022333) that are publicly accessible at https://ngdc.cncb.ac.cn/gsa. Additional datasets generated and/or analyzed during this study are available from the corresponding author upon request.
