# Supplementary material for: Single Cell and Spatial Transcriptomics Define a Proinflammatory and Profibrotic Niche After Kidney Injury
Source: Adv Sci (Weinh). 2025 Oct 3;13(2):e03691. doi: 10.1002/advs.202503691 (PMC12786295; doi:10.1002/advs.202503691)
Supplement: Supplementary file 1 — Supporting Information [file ADVS-13-e03691-s001.pdf]

# Single cell and spatial transcriptomic analyses define a proinflammatory and profibrotic niche after kidney injury

Li Li[#*1,2], Jinlin Liao[#1,2], Yuxi Zhang[#1,2], Zifu Yao[1], Junxin Huang[1], Kejia Wu[1], Lu Li[3], Yiling Peng[1], Haili Zhu[1], Xue Hong[1], Xi Liu[1], Lili Zhou[1], Fan Fan Hou[1,2], Haiyan Fu[*1,2] and Youhua Liu[*1,2]

[1]State Key Laboratory of Organ Failure Research, National Clinical Research Center of Kidney Disease, Division of Nephrology, Nanfang Hospital, Southern Medical University, and [2]Guangdong Provincial Institute of Nephrology, Guangzhou, China; [3]Department of Radiation Oncology, Nanfang Hospital, Southern Medical University, Guangzhou, China.

[#]These authors contributed equally.

[*]To whom correspondence should be addressed:

Youhua Liu, Ph.D, or Haiyan Fu, MD/Ph.D, or Li Li, Ph.D, Division of Nephrology, Nanfang Hospital, Southern Medical University, 1838 North Guangzhou Avenue, Guangzhou, 510515, China.

E-mail: liuyh@smu.edu.cn, or hy_fu426@126.com, or guilinlily3@i.smu.edu.cn.

## Detailed Methods

### Animal models

Mice with deficiency of TLR4 (*Tlr4*$^{-/-}$) in C57BL/10ScNJ background were purchased from GemPharmatech (Nanjing, China). The other animals were obtained from the Southern Medical University Animal Center in Guangzhou, China. Male BALB/c mice underwent unilateral ischemia-reperfusion injury (UIRI), and at day 10 post-UIRI, the contralateral intact kidney was removed.[1] The mice were euthanized 11 days after UIRI, and serum and kidney tissues collected for various analyses. For inhibition of TLR4 signaling, mice were daily injected intraperitoneally with TAK-242 at 8 mg/kg body weight from day 4 after UIRI, and sacrificed at 11 days. Bone marrow chimera models were established by transplanting the WT bone marrow to WT mice, or TLR4 KO bone marrow to WT mice. Mice were irradiated at a single dose of 1100 Rads and then underwent bone marrow transplantation. After 8 weeks of successful transplantation, a unilateral ischemia-reperfusion (UIRI) model was established. All animal experiments were conducted with approved protocols by the Experimental Animal Committee at Nanfang Hospital, Southern Medical University.

### Preparation of kidney tissue scaffold

The decellularized kidney tissue scaffold (dc-KTS) was prepared according to a well-established protocol reported previously.[2] At 21 days after UUO, kidneys from Tibet mini-pigs were arterially perfused in situ using pure water and phosphate-buffered saline (PBS) to remove the blood. The kidneys were then sliced to a uniform thickness. These kidney slices underwent a series of decellularization procedures using deoxyursocholic acid and Triton X-100. Macrophages were subsequently seeded onto the dc-KTS and incubated for 2 days. In some experiments, macrophages were inoculated onto the dc-KTS derived from different groups of mice for 2 days. Cell lysates were then prepared and analyzed by Western blot analyses.

### Cell culture and treatment

The culture of bone marrow-derived macrophages (BMDMs) was conducted as previously described.[3] In brief, femurs from adult mice aged 6-8 weeks were dissected to extract the bone

marrow, which was subsequently cultured in Dulbecco's modified Eagle's medium (DMEM) supplemented with 20% fetal bovine serum (FBS) and 30% L929 conditional medium to induce BMDM differentiation. The purity of BMDMs was assessed by staining with CD11b and F4/80 antibodies, with a threshold of >95% $CD11b^{+}F4/80^{+}$ population. Macrophages were incubated with TNC at different dosages for various periods of time as indicated. For some experiments, macrophages were pretreated with TAK-242 (5 nM), followed by incubating with vehicle or TNC (100 ng/ml).

**RNA-seq analysis**

Total RNA of the kidneys or cells was extracted with Trizol (Invitrogen). The RNA samples were assessed based on the A260/A280 absorbance ratio with a Nanodrop ND-2000 system (Thermo Scientific, USA), and the RNA integrity number (RIN) was determined by an Agilent Bioanalyzer 4150 system (Agilent Technologies, CA, USA). Paired-end libraries were prepared using an ABclonal mRNA-seq Lib Prep Kit (ABclonal, China) according to the manufacturer's instructions. RNA-seq was carried out on Novaseq 6000 (illumina) and MGISEQ-T7 (BGI), and 150 bp paired-end reads were generated. Each RNA-seq read was mapped to the mouse genome using HISAT2 software (http://daehwankimlab.github.io/hisat2/) with default values for the parameters.[4] FeatureCounts (http://subread.sourceforge.net/) was used to count the reads numbers mapped to each gene. The FPKM (fragments per kilobase per million mapped reads) of each gene was then calculated based on the length of the gene and reads count mapped to this gene. Differential expression analysis was performed using the DESeq2 (http://bioconductor.org/packages/release/bioc/html/DESeq2.html), adjusted $P < 0.05$ was considered as a significantly differential expression.

**Bioinformatics analyses for RNA-seq**

We used the R software package clusterProfiler[5] for the gene ontology (GO) and Kyoto Encyclopedia of genes and genomes (KEGG) enrichment analysis with the help provided by Shanghai Applied Protein Technology (Shanghai, China) and Guangzhou YanCeGene Co.Ltd. Enrichment analyses were applied based on the Fisher' exact test, when $P < 0.05$, it was considered

that the GO and KEGG functions are significantly enriched. Gene set enrichment analysis (GSEA) was performed by GSEA software with default parameters against KEGG gene sets.

**Single cell transcriptome sequencing and data processing**

1. Animal models for sequencing

Single-cell and spatial transcriptomic data were obtained from male C57BL/6 mice, weighing an approximate 20-22 grams each. The unilateral ischemia-reperfusion injury (UIRI) model was established, involving the use of microvascular aneurysm forceps to occlude the renal pedicle of the left kidney for 35 minutes, which induced ischemia-reperfusion injury. During this ischemic phase, a temperature-controlled heating system was implemented to preserve a consistent body temperature between 37-38°C. Upon removal of the forceps, we visually verified successful renal reperfusion. Ten days following the UIRI induction, the entire left kidney was carefully extracted via a left-side abdominal incision, with the retrieved kidney being utilized for consequent cell dissociation and section preparations. As a counter measure, sham operations were executed for the control group.

2. Cell dissociation

Fresh kidney tissue was rinsed in sterile PBS and diced into $1mm^3$ sections. The pieces were washed and immersed in a collagenase buffer at 37°C for 30 minutes to aid digestion. The reaction was halted with equal parts cold PBS/10% FBS. Next, the sample was filtered to exclude undigested tissue and the filtered cell solution was centrifuged. The recovered cells were suspended in serum-free medium, then stained with Acridine Orange and Propidium Iodide for viability assessment via automated counting.

3. Sequencing

Following manual quantification of our prepared cell suspension, we employed the 10x Genomics platform for transcriptomic sequencing. The procedure involved three primary steps: 1) cDNA Amplification: Within the Chromium system, a mix of cells, enzymes, and oil underwent emulsification. Subsequent addition of specific reagents facilitated reverse transcription and cDNA amplification. 2) Library Construction: We processed a portion of dsDNA with fragmentation enzymes, attached sequencing adapters, and amplified the mixture using PCR, culminating in the purification and quantification of the resulting PCR products. 3) Sequencing: Libraries with unique

indices were pooled for sequencing on the Illumina NovaSeq system.

4. Data processing using cell ranger software

We utilized the CellRanger's pipelines (version 7.1.0 - https://www.10xgenomics.com), tailoring procedures for Chromium Single Cell Gene Expression data, in which sequence alignment, UMI counting and cell barcode identification were integral, ensuring an accurate interpretation and further analysis of single-cell data.

5. Quality control and clustering analysis

We carried out single-cell data analysis and visualization utilizing Seurat,[6] SCP (https://github.com/zhanghao-njmu/SCP), and other R packages. Quality control standards were applied, filtering out cells that expressed genes < 1200 or > 7000, or cells with mitochondrial gene percentages > 30%, or ribosomal genes > 30%. We employed SoupX (version 1.6.2) to eliminate environmental RNA contamination and calculate cell expression profiles post background correction, utilizing standard parameters.[7] Post background correction, the gene expression profiles were subjected to another round of quality control. DoubletFinder (Version 2.0.3) was put to use to identify and expel heterotypic doublet peaks.[8] The double cell ratio (nEXP) was approximated in reference to the multiplet rate offered by 10X Genomics, also considering the quantity of each sample. There were, in total, 30,084 high-quality cells conserved for further analysis (nSham_1 = 5273, nSham_2 = 6578, nUIRI_1 = 9285, nUIRI_2 = 8948).

Normalization and scaling of gene expression were executed using NormalizeData and ScaleData functions, respectively. The foremost 2000 differential genes were chosen using FindVariableFeatures for PCA analysis. Dimension reduction was performed on the initial 20 principal components using RunPCA. Batch effects were mitigated with R package harmony(version 1.1.0)[9], and clustering was conducted with the FindClusters function at a resolution of 0.8.

Additionally, we supplemented our analysis with UUO samples from studies by other groups,[10-16] and IRI samples from Aggarwal et al.,[17] with detailed sample information provided in Supplementary Table 1. For UUO samples, we retained cells with 400–6000 expressed genes, fewer than 15,000 UMIs, <10% hemoglobin genes, <30% mitochondrial genes, and gene complexity (defined as log10(nFeature_RNA) / log10(nCount_RNA)) >0.8; for IRI samples, cells with 400–7000 expressed genes, <30% mitochondrial genes, <30% ribosomal genes, and gene

complexity >0.8 were retained.

6. Downstream analysis

(1) Differential gene analysis: We utilized the FindAllMarkers function in Seurat to discover differentially expressed genes (DEGs) across all clusters.[18] The 'Wilcox' test was applied, and we set the adjusted *P*-value at 0.05 and the log-fold change at log2(1.5). The FindMarker function was used to identify DEGs between cell clusters specified by ident.1 and ident.2, setting a minimum cell expression percentage (minpct) of 0.25 for at least one cluster.

(2) Enrichment analysis: ①We employed the clusterProfiler[5] (version 4.8.3) R package to conduct Kyoto Encyclopedia of Genes and Genomes (KEGG) pathway enrichment analysis on the DEGs, with a significance threshold set at a pvalue of 0.05. ②Gene Set Enrichment Analysis (GSEA) was performed via the gseKEGG function from the R package enrichplot (version 1.20.3) (https://github.com/YuLab-SMU/enrichplot). We set minimum and maximum gene sizes to 5 and 500 respectively. The Benjamini & Hochberg method was employed for p-value adjustments, with a cutoff of 0.4. ③Gene Set Variation Analysis (GSVA) was conducted utilizing the GSVA package (version 1.48.3). Gene sets were retrieved from the msigdbr package (version 7.5.1) (C2 for category and CP: KEGG for subcategory). The 'gsva' function was used in the enrichment analysis, with 'kcdf' set to "Poisson". We utilized the R package limma (version 3.56.2) for differential pathway analysis with adjustment using Holm's method. Only results with a *P*.Value <= 0.1 were deemed substantial.

(3) Cell-cell interaction analysis: Intercellular communication within the dataset was illuminated through the application of CellChat, a robust and specialized tool crafted specifically for this task. We initialized a CellChat object using the Seurat object and embedded cell-type information as metadata. CellChat operates based on its intrinsic database of ligand-receptor pairs, which encompasses three major categories of intercellular communication: Cell-Cell Contact, ECM-Receptor, and Secreted Signaling. We enriched this database with Tnc-associated pairs from literature.[19-22] The full list of these additional pairs is provided in Supplementary Table 4. CellChat then calculates a probability value ('prob') for each cell group's overexpressed ligand-receptor pairs, quantifying how likely they mediate communication between cell groups. We consider communication with prob values over 0.01 significant. In the resultant bubble plot, communication intensity is depicted using the transform of log10(prob) + 1, falling within the range of 0 and 1.

(4) Cell type correlation analysis: To evaluate the consistency of cell type annotations between our dataset and a published IRI scRNA-seq dataset, we conducted a cross-dataset correlation analysis. First, the intersection of expressed genes was identified between the two datasets. For each cell type, the average expression levels of the shared genes were calculated. Pearson correlation coefficients were then computed to assess the similarity in gene expression profiles between corresponding cell types. The analysis was performed using the psych (version 2.4.12) R package, and the results were visualized as a correlation heatmap.

**Spatial Transcriptomics Sequencing and Data Processing**

**Visium data processing**

1. Tissue sectioning

Mouse kidney samples stored in a frozen state were embedded with OCT compound (Tissue-Tek 4583) and solidified on dry ice. Post solidification, sections were stored at -80℃. For the preparation of sections for Visium Spatial Transcriptomics sequencing, samples were equilibrated at -18℃ and a 10μm thick section was cut onto the active sequencing area (6mm x 6mm) of a spatial barcoded slide. After sectioning, the slides were placed in a sealed container and stored at -80℃ until the time of spatial library construction. Furthermore, H&E staining and photographic imaging were performed on the frozen slices in the specific capture area on the slide to retain histomorphological information of the sample. Under specified conditions, the fixed sections underwent tissue clearing to remove lipids and other interfering substances in the tissues for subsequent mRNA capture and analysis.

2. cDNA synthesis and library construction

Primers with spatial barcodes were used to capture mRNA released from cleared tissue. The captured mRNA reacted with the primers to synthesize the corresponding cDNA, which was then amplified by PCR. The PCR-amplified products were quality checked to ensure they met sequencing requirements. Upon successful verification, they underwent library construction. Initially, fragmentation of the product was performed, followed by end repair and A-tailing. The repaired fragments underwent screening to select the appropriate fragment size. Next was adapter ligation, where sequencing adapters were attached to the ends of the repaired fragments. Dual indexes were introduced through SI-PCR amplification, and after fragment screening, the final cDNA library was obtained.

3. Sequencing

Sequencing was carried out using the Illumina NovaSeq S4 sequencing platform. Dual index sequencing was performed in accordance with the guidelines of 10x Genomics Visium manufacturer (PN-1000185, lot number 155614, revision D), with the goal of obtaining 125 million reads. The raw data (FASTQ format) obtained from sequencing were aligned with the mm10 reference genome using appropriate alignment software (such as Bowtie, STAR, etc.), manually aligned with the corresponding Hematoxylin and Eosin-stained images, and spatial information was associated with gene expression data. Normalization was performed using the 10x Genomics Space Ranger count software (Spatial 3' v1; spaceranger-1.2.1). All time-point sample sequencing libraries were integrated using agg to consolidate data from different time points. Post normalization, approximately 18,000-21,000 unique genes were identified for each sample.

4. Upstream analysis

The raw sequencing data (FASTQ files) and H&E stained images of the tissue sections were input into Space Ranger software. A comparison of raw data with the reference genome was performed, and tissue detection was carried out to obtain tissue structure information. Baseline calibration and barcode/UMI statistics were applied to gene expression data for sequencing depth correction and experimental quality assessment. Finally, web_summary reports, spot-gene expression matrices, etc. were generated to summarize experimental quality, provide gene expression data, and visualize spatial information.

5. Downstream analysis

(1) Data preprocessing: Spatial transcriptomics (ST) data were preprocessed using the R package Seurat (version 5.3.0) and Semla (version 1.3.1).[23] For the IRI spatial transcriptomics data, only spots with 'nfeature' greater than 1000 were retained for further processing. Normalization was performed using the NormalizeData function, and highly variable genes were identified using the FindVariableFeatures function. Data integration was conducted using Seurat’s anchor-based workflow, including FindIntegrationAnchors and IntegrateData. Principal component analysis (PCA) was conducted using the RunPCA function, selecting the top 10 principal components. Clustering was performed using the FindClusters function with a resolution parameter set to 1.2. For the identification of DEGs, we employed the FindAllMarkers and FindMarkers functions, while spatially

variable features were detected using the FindSpatiallyVariableFeatures function. For the UUO spatial transcriptomics data (derived from the study by Y. Yasui et al., 202411; see Supplementary Table 1 for detailed sample information) the same preprocessing workflow was applied.[12] The only differences were the use of the top 20 principal components for RunPCA and a clustering resolution of 0.8.

(2) Enrichment analysis: An in-depth enrichment analysis was performed maintaining consistency with the scRNA-seq method section described previously.

(3) Spatial transcriptomics deconvolution: The deconvolution was performed using spacexr package (version 2.2.1), based on the Robust Cell Type Decomposition (RCTD), a supervised learning method suitable for deconstructing the RNA sequence mixtures in spatial spots.[24] By following the official tutorial, we instantiated the RCTD object via create.RCTD function and executed the core computations using the run.RCTD function. The 'doublet_mode' parameter was set to "full", enabling the model to account for contributions from singlets, doublets, and triplets, thereby improving the resolution of complex cellular mixtures.

(4) NMF: 1) NMF calculation: Non-negative matrix factorization (NMF) was applied to the integrated spatial transcriptomic data using the RunNMF function from the Singlet (version 0.99.8) R package. The optimal number of factors was determined using the RankPlot function. To spatially map NMF results to corresponding tissue samples, metadata and NMF embedding matrices were aligned and merged into a unified Semla object. The feature loadings and embeddings derived from NMF were stored in the misc slot of the merged object for downstream analysis. 2) Correlation analysis between NMF factors and RCTD deconvolution scores: To assess the correspondence between NMF components and predicted cell type compositions, we computed the correlation between NMF factor embeddings and RCTD-inferred cell type proportions. Specifically, we extracted the normalized cell type weights from the RCTD output and matched them with NMF embeddings across shared spatial spots. Spearman correlation coefficients were calculated to quantify the association between each NMF component and cell type.

(5) Calculation of gene expression correlation: We utilized Pearson correlation coefficient to measure the correlation between genes at spatial locations. Subsequently, we selected the top 10 molecules with the highest correlation to the target gene for further analysis. To ensure the statistical

significance of the results, we set a threshold for the $P$-value at 0.01.

**(6)** Cell-cell interaction analysis: To investigate intercellular communication between spatially defined regions, we applied CellChat (version 2.1.0)[25] to spatial transcriptomic datasets. A CellChat object was initialized from the Seurat object, with region-level annotations embedded as metadata. The analysis was performed using the CellChat ligand–receptor interaction database, which includes three major signaling categories: Cell–Cell Contact, ECM–Receptor, and Secreted Signaling. We additionally incorporated Tnc-associated ligand–receptor pairs curated from the literature, consistent with those used in our single-cell analysis. Spatial proximity was modeled by enabling distance.use and setting interaction.range = 250, limiting interactions to within 250 pixels. Communication probability was estimated using type = "truncatedMean" with trim = 0.1, reducing the influence of outlier values. All other parameters followed the default spatial analysis pipeline recommended by CellChat.

**Visium HD data processing**

1. Tissue Sectioning

Formalin-fixed paraffin-embedded (FFPE) kidney tissue blocks were obtained from C57BL/6 male mice (8 weeks old) subjected to unilateral ischemia-reperfusion injury (UIRI) for 10 days, consistent with the animal model used in the preceding scRNA-seq and 10x Visium experiments. Serial 5 μm sections were prepared from the FFPE blocks using a Leica RM2255 microtome, following the Visium HD Spatial Gene Expression FFPE Tissue Preparation Guide (10x Genomics, CG000684 Rev A), and mounted onto Sigma-Aldrich Poly Prep Slides. The slides were air-dried overnight at room temperature and then incubated at 60°C for 2 hours. Subsequently, tissue sections underwent deparaffinization, hematoxylin and eosin (H&E) staining, and brightfield imaging using a Leica Aperio Versa 8 microscope, strictly adhering to the 10x Genomics Visium HD Spatial Gene Expression FFPE protocol, in order to preserve histomorphological features for spatial transcriptomic analysis.

2. cDNA Synthesis, Library Construction, and Sequencing

Next, the Mouse Whole Transcriptome Probe Panel was applied to the tissue sections. After hybridization of probes to target mRNA sequences and subsequent ligation, the slides underwent

RNase treatment and tissue permeabilization using the Visium HD instrument. The ligated probe complexes were then captured by spatially barcoded oligonucleotides embedded within the active capture areas of the slide, enabling the generation of high-resolution spatial transcriptomic libraries. These libraries were subsequently sequenced on an Illumina platform according to the manufacturer’s protocol.

3. Upstream Data Analysis

After cDNA library construction and high-throughput sequencing, raw sequencing data underwent initial quality control using in-house scripts, which included assessment of overall data quality and evaluation of GC content across sequencing cycles. Subsequently, FASTQ files and histological images were analyzed using the FFPE 'count' pipeline in 10x Genomics Space Ranger software (v3.1), where sequencing reads were aligned to the mouse reference genome (mm10) using a short-read probe alignment algorithm.

4. Downstream analysis：

(1) Data preprocessing: High-resolution spatial transcriptomic data (Visium HD) from control and UIRI10D kidney tissue samples were subjected to integrated analysis using R packages including Semla and Seurat. Control group data were obtained from the official Visium HD dataset (Mouse kidney FFPE tissue section (C57BL/6, male, 8 weeks), Visium HD Spatial Gene Expression, Probe Set v2.0, sequenced on Illumina NovaSeq 6000 (43 bp × 50 bp reads), processed with Space Ranger v3.0, 10x Genomics (2024, June 12). https://www.10xgenomics.com/datasets/visium-hd-cytassist-gene-expression-libraries-of-mouse-kidney), while UIRI 10D samples were generated using the same modeling protocol as described previously for single-cell and standard spatial transcriptomic experiments. After initial preprocessing, Semla objects from control and UIRI 10D samples were merged. In the merged object, each spatial layer was independently normalized (NormalizeData), scaled (ScaleData), and subjected to highly variable feature selection (FindVariableFeatures). Dimensionality reduction was performed using principal component analysis (RunPCA). To enable cross-sample integration, canonical correlation analysis (CCA) was applied using the IntegrateLayers function. Clustering analysis was performed using the top 30 principal components with a resolution of 0.8.

(2) Spatial transcriptomics deconvolution and NMF: Spatial transcriptomic deconvolution for

Visium HD data was performed using the same pipeline as that for standard Visium data. The only modification was the setting of ‘doublet_mode’ to ‘doublet’ due to the smaller spot size and fewer cell types per spot in Visium HD data. Non-negative matrix factorization (NMF) analysis for Visium HD data was conducted using the same computational framework as applied to standard Visium datasets, without modification to algorithmic parameters or implementation strategy.

**Knockdown of TNC in vivo**

The knockdown of TNC expression in vivo was conducted by using an shRNA-mediated approach, as previously reported.[26] Male BALB/c mice were divided into three groups, each consisting of six animals: (1) mice subjected to sham operation, (2) UIRI mice administered with control shRNA, and (3) UIRI mice administered with TNC-shRNA. Mice were subjected to tail-vein injections of either pLVX-shTNC or control (pLVX-control) plasmids at 4 days after UIRI.

**EdU incorporation assay**

The 5-ethynyl-2'-deoxyuridine (EdU) incorporation assay was conducted according to a routine protocol. Briefly, cells were seeded onto six-well plates and treated with TNC as indicated. Subsequently, the cells were incubated with EdU (10 μM) for 6 h, and then fixed. They were then incubated with the EdU reaction mixture for 30 min at room temperature. To visualize the stained samples, Hoechst reaction solution was added to each well. The stained samples were observed using an Eclipse E600 epifluorescence microscope equipped with a digital camera (Nikon, Tokyo, Japan).

**Flow cytometry analysis**

Macrophages were incubated with TNC for 48 h. Subsequently, they were rinsed with cold PBS and stained using the Cycletest Plus DNA Reagent Kit (Becton Dickinson, CA, USA) according to the manufacturer's guidelines. The cell cycle distribution was assessed using a FACSCanto II Flow cytometry (Becton Dickinson, CA), and the data were analyzed by using the ModFit LT3.3 software. A total of 10,000 events per sample were recorded for each experimental trial. For analyzing mouse kidney macrophages, single-cell suspensions from the kidneys were made according to an established protocol.[27] After washing with cold PBS, cells were stained with fluorescent-conjugated antibodies

(F4/80-PE, CD11b-FITC, CD45-APC). Acquisition was performed on a FACSCanto II Flow cytometry (Becton Dickinson, CA, USA). Analysis was performed using the FlowJo software.

**Macrophage phagocytosis experiment**

Phagocytosis was detected through flow cytometry as previously reported.[28] Macrophages were incubated with TNC for 48 h, and fluorescent microspheres labeled with green fluorescent protein (GFP) were then added. After incubation for 1 h, cells were collected for flow cytometry detection. Analysis was performed using the FlowJo software.

**Transwell migration assay**

The macrophage migration assay was performed using 24-well Transwell chambers (Corning). Macrophages were incubated with TNC and cultured in the upper chamber for 48 h. The migratory capacity was assessed by quantifying the number of cells that traversed the membrane after staining with hematoxylin. Five fields were randomly chosen and examined under an inverted microscope. The results were averaged for each sample, and the experiment was replicated three times.

**Western blot analyses**

Protein expression was analyzed by Western blot analysis as described previously.[29, 30] The antibodies used are listed in Supplementary Table 2.

**Quantitative real-time RT-PCR**

Total RNA was isolated and qPCR analysis was performed using an ABI PRISM 7000 Sequence Detection System, as previously described.[2] The mRNA levels of various genes were determined by normalizing with β-actin. The sequences of primer pairs are listed in Supplementary Table 3.

**Histology, immunohistochemical and immunofluorescence staining**

Paraffin-embedded sections of mouse kidneys were prepared using a standard procedure. Masson's trichrome staining (MTS) reagents were employed to stain the sections. Immunohistochemical and immunofluorescence staining were conducted by following previously established protocols.

Antibodies used are listed in Supplementary Table 2.

**Statistical analyses**

All data examined were expressed as mean ± SEM. Statistical analyses of the data were performed using SPSS Statistics (SPSS Inc, Chicago, IL). Group comparisons were made through t test, or by employing one-way ANOVA followed by Fisher's least significant difference test or Dunnett T3 test. $P < 0.05$ was considered significant.

**Reference**


1. Li L, Liao J, Yuan Q, Hong X, Li J, Peng Y, He M, Zhu H, Zhu M, Hou FF *et al.* Fibrillin-1-enriched microenvironment drives endothelial injury and vascular rarefaction in chronic kidney disease. *Sci Adv* **2021**, 7(5):eabc7170.
2. Li L, He M, Tang X, Huang J, Li J, Hong X, Fu H, Liu Y. Proteomic landscape of the extracellular matrix in the fibrotic kidney. *Kidney Int* **2023**, 103(6):1063-1076.
3. Xing J, Weng L, Yuan B, Wang Z, Jia L, Jin R, Lu H, Li XC, Liu YJ, Zhang Z. Identification of a role for TRIM29 in the control of innate immunity in the respiratory tract. *Nat Immunol* **2016**, 17(12):1373-1380.
4. Kim D, Langmead B, Salzberg SL. HISAT: a fast spliced aligner with low memory requirements. *Nat Methods* **2015**, 12(4):357-360.
5. Yu G, Wang LG, Han Y, He QY. clusterProfiler: an R package for comparing biological themes among gene clusters. *Omics* **2012**, 16(5):284-287.
6. Hao Y, Stuart T, Kowalski MH, Choudhary S, Hoffman P, Hartman A, Srivastava A, Molla G, Madad S, Fernandez-Granda C *et al.* Dictionary learning for integrative, multimodal and scalable single-cell analysis. *Nat Biotechnol* **2024**, 42(2):293-304.
7. Young MD, Behjati S. SoupX removes ambient RNA contamination from droplet-based single-cell RNA sequencing data. *Gigascience* **2020**, 9(12).
8. McGinnis CS, Murrow LM, Gartner ZJ. DoubletFinder: Doublet Detection in Single-Cell RNA Sequencing Data Using Artificial Nearest Neighbors. *Cell Syst* **2019**, 8(4):329-337.e324.
9. Korsunsky I, Millard N, Fan J, Slowikowski K, Zhang F, Wei K, Baglaenko Y, Brenner M, Loh PR, Raychaudhuri S. Fast, sensitive and accurate integration of single-cell data with Harmony. *Nat Methods* **2019**, 16(12):1289-1296.
10. Xu Y, Zheng Z, Oswald MS, Cheng G, Liu J, Zhai Q, Kruegel U, Schaefer M, Gerhardt H, Endlich N *et al.* Single-Cell RNA Sequencing Delineates Renal Anti-Fibrotic Mechanisms Mediated by TRPC6 Inhibition. *Adv Sci (Weinh)* **2025**:e01175.
11. Youssef KK, Narwade N, Arcas A, Marquez-Galera A, Jiménez-Castaño R, Lopez-Blau C, Fazilaty H, García-Gutierrez D, Cano A, Galcerán J *et al.* Two distinct epithelial-to-mesenchymal transition programs control invasion and inflammation in segregated tumor cell populations. *Nat Cancer* **2024**, 5(11):1660-1680.
12. Yasui Y, Murata T, Tsuboi Y, Murai A, Horiba N. CH6824025, Potent and Selective Discoidin Domain Receptor 1 Inhibitor, Reduces Kidney Fibrosis in Unilateral Ureteral Obstruction Mice. *J Pharmacol Exp Ther* **2024**, 391(3):450-459.
13. Rudman-Melnick V, Vanhoutte D, Stowers K, Sargent M, Adam M, Ma Q, Perl AKT, Miethke AG, Burg A, Shi T *et al.* Gucy1α1 specifically marks kidney, heart, lung and liver fibroblasts. *Sci Rep* **2024**, 14(1):29307.
14. Rudman-Melnick V, Adam M, Stowers K, Potter A, Ma Q, Chokshi SM, Vanhoutte D, Valiente-Alandi I, Lindquist DM, Nieman ML *et al.* Single-cell sequencing dissects the transcriptional identity of activated fibroblasts and identifies novel persistent distal tubular injury patterns in kidney fibrosis. *Sci Rep* **2024**, 14(1):439.
15. O'Sullivan ED, Mylonas KJ, Bell R, Carvalho C, Baird DP, Cairns C, Gallagher KM, Campbell R, Docherty M, Laird A *et al.* Single-cell analysis of senescent epithelia

reveals targetable mechanisms promoting fibrosis. *JCI Insight* **2022**, 7(22).
16. Conway BR, O'Sullivan ED, Cairns C, O'Sullivan J, Simpson DJ, Salzano A, Connor K, Ding P, Humphries D, Stewart K *et al*. Kidney Single-Cell Atlas Reveals Myeloid Heterogeneity in Progression and Regression of Kidney Disease. *J Am Soc Nephrol* **2020**, 31(12):2833-2854.
17. Aggarwal S, Wang Z, Rincon Fernandez Pacheco D, Rinaldi A, Rajewski A, Callemeyn J, Van Loon E, Lamarthée B, Covarrubias AE, Hou J *et al*. SOX9 switch links regeneration to fibrosis at the single-cell level in mammalian kidneys. *Science* **2024**, 383(6685):eadd6371.
18. Butler A, Hoffman P, Smibert P, Papalexi E, Satija R. Integrating single-cell transcriptomic data across different conditions, technologies, and species. *Nat Biotechnol* **2018**, 36(5):411-420.
19. Midwood KS, Hussenet T, Langlois B, Orend G. Advances in tenascin-C biology. *Cell Mol Life Sci* **2011**, 68(19):3175-3199.
20. Yilmaz A, Loustau T, Salomé N, Poilil SS, Li C, Tucker RP, Izzi V, Lamba R, Koch M, Orend G. Advances on the roles of tenascin-C in cancer. *J Cell Sci* **2022**, 135(18).
21. Kimura T, Tajiri K, Sato A, Sakai S, Wang Z, Yoshida T, Uede T, Hiroe M, Aonuma K, Ieda M *et al*. Tenascin-C accelerates adverse ventricular remodelling after myocardial infarction by modulating macrophage polarization. *Cardiovasc Res* **2019**, 115(3):614-624.
22. Midwood KS, Chiquet M, Tucker RP, Orend G. Tenascin-C at a glance. *J Cell Sci* **2016**, 129(23):4321-4327.
23. Larsson L, Franzén L, Ståhl PL, Lundeberg J. Semla: a versatile toolkit for spatially resolved transcriptomics analysis and visualization. *Bioinformatics* **2023**, 39(10).
24. Cable DM, Murray E, Zou LS, Goeva A, Macosko EZ, Chen F, Irizarry RA. Robust decomposition of cell type mixtures in spatial transcriptomics. *Nat Biotechnol* **2022**, 40(4):517-526.
25. Jin S, Plikus MV, Nie Q. CellChat for systematic analysis of cell-cell communication from single-cell transcriptomics. *Nat Protoc* **2025**, 20(1):180-219.
26. Zhu H, Liao J, Zhou X, Hong X, Song D, Hou FF, Liu Y, Fu H. Tenascin-C promotes acute kidney injury to chronic kidney disease progression by impairing tubular integrity via αvβ6 integrin signaling. *Kidney Int* **2020**, 97(5):1017-1031.
27. Shinde R, Hezaveh K, Halaby MJ, Kloetgen A, Chakravarthy A, Da Silva Medina T, Deol R, Manion KP, Baglaenko Y, Eldh M *et al*. Apoptotic cell-induced AhR activity is required for immunological tolerance and suppression of systemic lupus erythematosus in mice and humans. *Nat Immunol* **2018**, 19(6):571-582.
28. Barkal AA, Brewer RE, Markovic M, Kowarsky M, Barkal SA, Zaro BW, Krishnan V, Hatakeyama J, Dorigo O, Barkal LJ *et al*. CD24 signalling through macrophage Siglec-10 is a target for cancer immunotherapy. *Nature* **2019**, 572(7769):392-396.
29. Li L, Lu M, Peng Y, Huang J, Tang X, Chen J, Li J, Hong X, He M, Fu H *et al*. Oxidatively stressed extracellular microenvironment drives fibroblast activation and kidney fibrosis. *Redox Biol* **2023**, 67:102868.
30. Yuan Q, Ren Q, Li L, Tan H, Lu M, Tian Y, Huang L, Zhao B, Fu H, Hou FF *et al*. A Klotho-derived peptide protects against kidney fibrosis by targeting TGF-β signaling.

*Nat Commun* **2022**, 13(1):438.

**Supplementary Tables**

**Supplementary Table S1. Publicly available single-cell and spatial transcriptomics datasets**

| Sequencing Technologies | GEO Sample | PMID | Mouse characteristics | | | Modeling methods |
|---|---|---|---|---|---|---|
| **scRNA-seq** | | | **Strain/Sex** | | **Age** | |
| | GSM5333084 | | | | | Sham |
| | GSM5333085 | 39414946 | C57BL/6J | male | 12 week | UUO 10D |
| | GSM5333086 | | | | | UUO 10D |
| | GSM8305766 | | | | | UUO 7D (treatment: Vehicle) |
| | GSM8305767 | 40525246 | C57BL/6 | / | / | UUO 7D (treatment: Vehicle) |
| | GSM8305768 | | | | | UUO 7D (treatment: Vehicle) |
| | GSM5953829 | | | | | Sham |
| | GSM5953830 | | | | | Sham |
| | GSM5953831 | | | | | Sham |
| | GSM5953832 | | | | | Sham |
| | GSM5953833 | 38172172<br>39592775 | C57BL/6 | male | 10 week | Sham |
| | GSM5953837 | | | | | UUO 28D |
| | GSM5953838 | | | | | UUO 28D |
| | GSM5953839 | | | | | UUO 28D |
| | GSM4151577 | 32978267<br>36509292 | C57BL/6 | male | 8 week | Sham |
| | GSM4151579 | | | | | UUO 7D |
| | GSM5904833 | | | | | IRI 10D |
| | GSM5904834 | 38386758 | C57BL/6 | male | 9 -12 week | IRI 10D |
| | GSM5904835 | | | | | IRI 10D |
| **Visium** | | | | | | |
| | GSM8440998 | | | | | UUO 7D (treatment: Vehicle) |
| | GSM8441001 | | | | | UUO 7D (treatment: Vehicle) |
| | GSM8441002 | 39379147 | C57BL/6J | male | / | UUO 7D (treatment: Vehicle) |
| | GSM8440997 | | | | | Sham (treatment: Vehicle) |

**Supplementary Table S2. The sources of antibodies used in this study**

| Antibodies | Catalogue number | Company | Location |
|---|---|---|---|
| **Primary antibodies** | | | |
| anti-TNC | Ab108930 | Abcam | Cambridge, MA |
| anti-TNC | T3413 | Sigma-Aldrich | St. Louis, MO |
| anti-c-Fos | BA0207-2 | Boster Biological Technology | Wuhan, China |
| anti-PCNA | sc-56 | Santa Cruz Biotechnology | Santa Cruz, CA |
| anti-c-Myc | 5605S | Cell Signaling Technology | Danvers, MA |
| anti-TLR4 | A5258 | Abclonal | Wuhan, China |
| anti-TLR4 | sc-293072 | Santa Cruz Biotechnology | Santa Cruz, CA |
| anti-p-P65 | 3033S | Cell Signaling Technology | Danvers, MA |
| anti-P65 | 4764S | Cell Signaling Technology | Danvers, MA |
| anti-TNF-α | Ab1793 | Abcam | Cambridge, MA |
| anti-iNOS | Ab15323 | Abcam | Cambridge, MA |
| anti-CCL2 | Ab8101 | Abcam | Cambridge, MA |
| anti-Mannose Receptor | Ab8918 | Abcam | Cambridge, MA |
| anti-Arginase-1 | GTX109242 | Genetex | San Antonio, TX |
| anti-fibronectin | F3648 | Sigma-Aldrich | St. Louis, MO |
| anti-α-SMA | A5228 | Sigma-Aldrich | St. Louis, MO |
| anti-F4/80 | 12-4801-80 | eBioscience | Santa Clara, CA |
| anti-F4/80 | MCA497 | Bio-Rad | Hercules, CA |
| anti-CD11b | 11-0112-41 | eBioscience | Santa Clara, CA |
| anti-CCR2 | Ab273050 | Abcam | Cambridge, MA |
| anti-α-Tubulin | RM2007 | Ray Antibody Biotech | Peachtree Corners, GA |
| **Secondary antibodies** | | | |
| Goat anti-mouse | BA1050 | Boster Biological Technology | Wuhan, China |
| Goat anti-rabbit | BA1054 | Boster Biological Technology | Wuhan, China |

**Supplementary Table S3. Nucleotide sequences of the primers used for qPCR**

| Mouse gene | Primer Sequence 5' to 3' | |
|---|---|---|
| | Forward | Reverse |
| *i-NOS* | GTTCTCAGCCCAACAATACAAGA | GTGGACGGGTCGATGTCAC |
| *TNF-α* | CGTAGCAAACCACCAAGTG | CCTTGAAGAGAACCTGGGAG |
| *IL-6* | TGGTCTTCTGGAGTACCATAGC | TCTGTGACTCCAGCTTATCTCTTG |
| *IL-1β* | GAAATGCCACCTTTTGACAGTG | TGGATGCTCTCATCAGGACAG |
| *β-actin* | AAGATCAAGATCATTGCTCCTCCTG | CGCAGCTCAGTAACAGTCCG |

**Supplementary Table S4. TNC-associated receptor-ligand pairs**

| Interaction_name | Pathway_name | Ligand | Receptor | Annotation | Interaction_name_2 |
|---|---|---|---|---|---|
| TNC_EGFR | TENASCIN | Tnc | Egfr | ECM-Receptor | Tnc - Egfr |
| TNC_CSPG5 | TENASCIN | Tnc | Cspg5 | ECM-Receptor | Tnc - Cspg5 |
| TNC_TLR4 | TENASCIN | Tnc | Tlr4 | ECM-Receptor | Tnc - Tlr4 |
| TNC_PTPRZ1 | TENASCIN | Tnc | Ptprz1 | ECM-Receptor | Tnc - Ptprz1 |
| TNC_SCN2B | TENASCIN | Tnc | Scn2b | ECM-Receptor | Tnc - Scn2b |
| TNC_ANXA6 | TENASCIN | Tnc | Anxa6 | ECM-Receptor | Tnc - Anxa6 |
| TNC_ITGA2_ITGB1 | TENASCIN | Tnc | ITGA2_ITGB1 | ECM-Receptor | Tnc - (Itga2+Itgb1) |
| TNC_ITGA7_ITGB1 | TENASCIN | Tnc | ITGA7_ITGB1 | ECM-Receptor | Tnc - (Itga7+Itgb1) |
| TNC_ITGAV_ITGB1 | TENASCIN | Tnc | ITGAV_ITGB1 | ECM-Receptor | Tnc - (Itgav+Itgb1) |

## Supplementary Figures

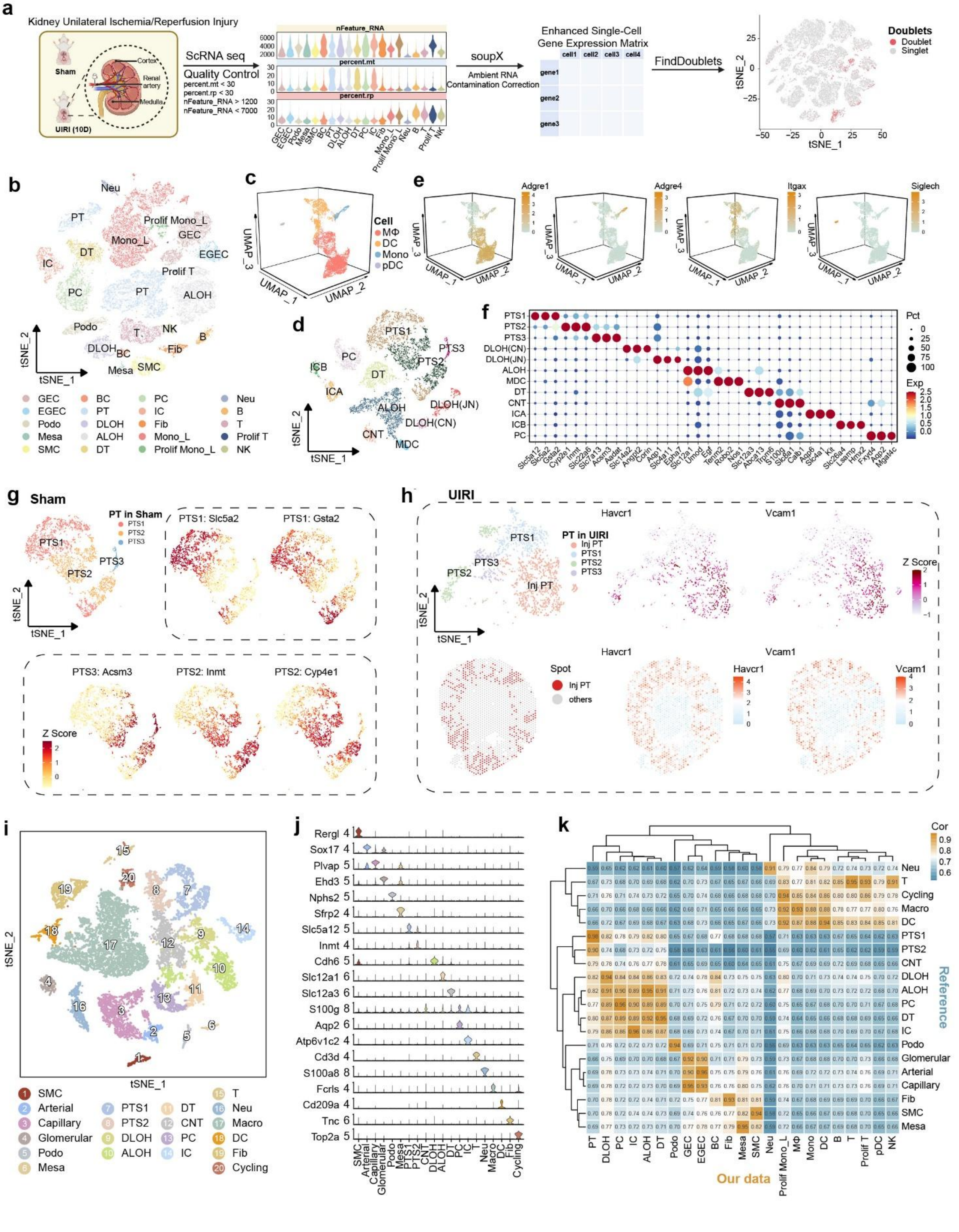


**Figure S1.** Quality control and spatial markers of major cell types. (a) Upstream analysis of

single-cell data encompasses quality control, ambient gene subtraction, and doublet detection. The quality control metrics are depicted using violin plots for the proportion of mitochondrial genes (percent.mt), ribosomal gene proportion (percent.rp), and the number of genes detected (nFeatures) per cell. The SoupX algorithm is employed to remove ambient RNA contamination, resulting in a refined gene expression matrix. After identification and removal of potential doublets using DoubletFinder, the purified dataset is processed for downstream analysis. (b) t-SNE visualization reveals the major cell types identified within our dataset. (c) 3D UMAP presentation of mononuclear lineage cells, including monocytes (Mono), macrophages (MΦ), dendritic cells (DC), and plasmacytoid dendritic cells (pDC). Uniform Manifold Approximation and Projection, UMAP. (d) t-SNE plot demonstrates the classification of normal renal cell populations. (e) 3D FeaturePlot highlights the expression of cell-type-specific markers within the mononuclear lineage cells. (f) Dot plot of renal subtypes' markers, with color depth showing expression (Exp) and size indicating the expression percentage (Pct) within each cell type. (g) t-SNE plot depicting proximal tubule (PT) subclusters, including S1, S2, S3 segments, with Featureplot showing PT subgroups' marker expression. (h) t-SNE plot shows UIRI group PT clustering, including injured proximal tubules (Inj PT). Featureplot demonstrates expression of Havcr1 (Kim-1) and Vcam1 in Inj PT (above). A spot cluster on spatial transcriptomics is annotated as Inj PT with spatial Featureplot demonstrating Havcr1 and Vcam1 expression patterns (below). (i) t-SNE plot depicts cellular distribution in IRI 10D kidney models using scRNA-seq data from Aggarwal et al, including arterial cells, capillary cells, and cycling cells. (j) Stacked violin plots show marker gene expression profiles across cell subpopulations in the Aggarwal dataset. (k) Correlation heatmap compares expression similarity between our data and Aggarwal's IRI 10D scRNA-seq data using Pearson coefficients.

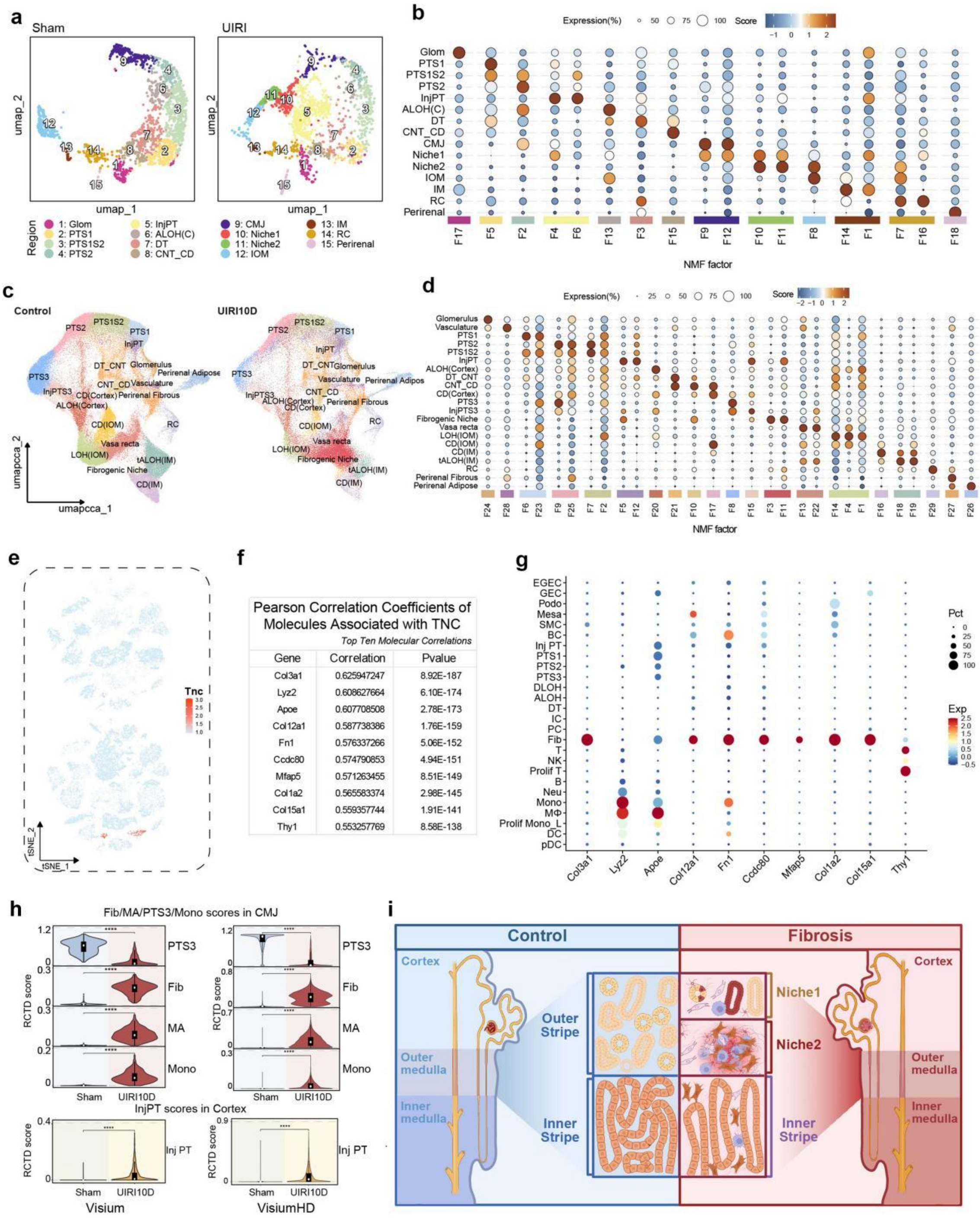


**Figure S2.** Integrated analysis of standard and HD Visium data in UIRI kidneys reveals injury-specific spatial architecture. (a) UMAP plot of integrated Visium data (Sham vs. UIRI kidneys). The distributions of major renal regions are largely consistent between samples, while InjPT cells and the fibrogenic niche are exclusively present in the UIRI kidney. (b) Bubble plot showing the association between spatial regions and NMF-derived factors based on standard Visium

spatial transcriptomics data. (c) UMAP plot of integrated Visium HD data (Sham vs. UIRI kidneys). (d) Bubble plot showing the association between spatial regions and NMF-derived factors based on Visium HD spatial transcriptomics data. (e) Single-cell Featureplot highlighting the expression patterns of Tnc in the sham and UIRI groups. (f) Pearson correlation coefficients and corresponding p-values for the top 10 genes most strongly associated with TNC expression. (g) Bubble plot showing the expression of the top 10 molecules most associated with TNC across single-cell types. (h) Violin plots comparing spatial distribution differences of cell types between Sham and UIRI groups ($^{**}$Wilcoxon test; $^{*}P <= 0.05$, $^{**}P <= 0.01$, $^{***}P <= 0.001$, $^{****}P <= 0.0001$). (Top) Fibroblasts, macrophages, monocytes, and PTS3 at the cortico-medullary junction (CMJ). (Bottom) Injured PT cells (Inj PT) in the cortical region. Left: Visium data; right: VisiumHD data. (i) Schematic illustrating the spatial relationship between fibrogenic niches (Niche1/Niche2) and classical renal anatomical compartments.

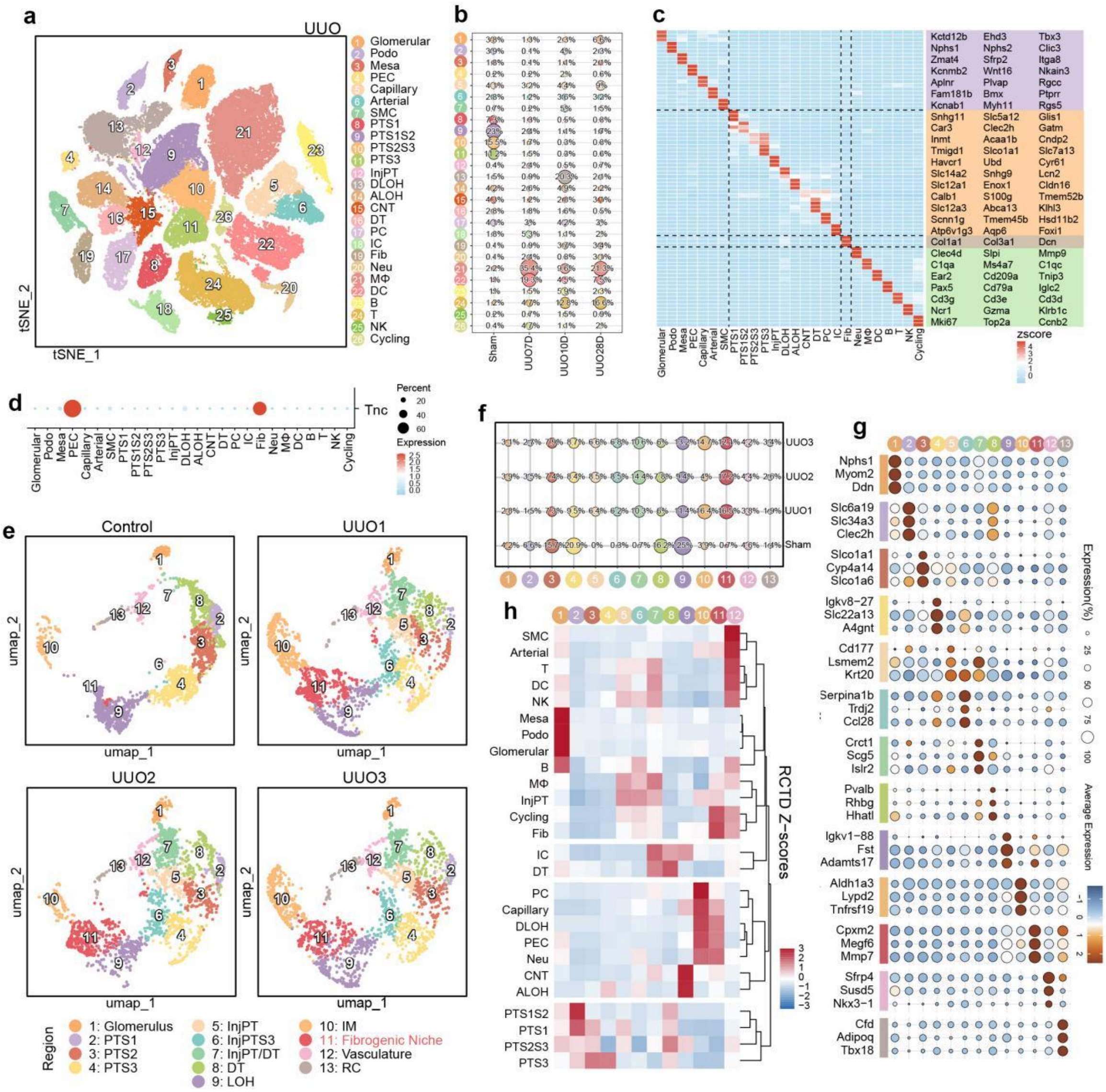


**Figure S3.** Integrated single-cell and spatial transcriptomic profiling of renal remodeling in UUO-induced kidney fibrosis. (a) tSNE plot of scRNA sequencing data from UUO kidney samples, illustrating the distribution of renal parenchymal and immune cell types. (b) Bubble plot illustrating the relative proportions of major renal cell types across scRNA sequencing samples from Sham and UUO groups at different time points. (c) A comprehensive heatmap depicting the unique marker genes signature of major renal cell types. (d) Bubble plot showing the expression levels of the Tnc gene across different cell types in UUO samples. (e) UMAP plot of integrated Visium data (Sham vs. UUO kidneys). (f) Bubble plot illustrating the relative proportions of major kidney cell types in Sham and UUO samples. (g) Bubble plot depicting the expression patterns of marker genes across distinct renal cell regions in spatial transcriptomics data. (h) A heatmap showing the deconvolution scores of cell type compositions across different regions in Visium spatial transcriptomics data, obtained using

the RCTD method.

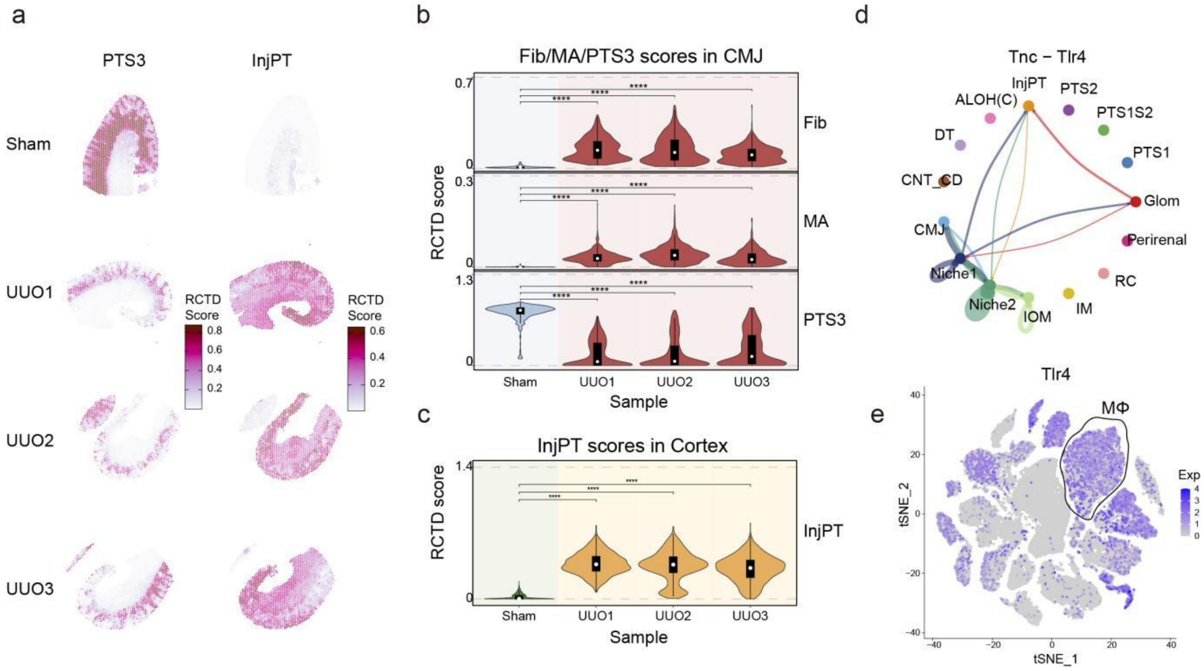


**Figure S4**. Spatial characterization of fibrogenic niches in kidney injury models. (a) Spatial FeaturePlots illustrating RCTD-derived scores for PTS3 and InjPT cell types in the Sham and UUO groups. (b) Violin plots comparing fibroblasts, macrophages, and PTS3 spatial distribution between Sham and UIRI groups at the cortico-medullary junction (CMJ) (Wilcoxon test, $^{*}P \leq 0.05$, $^{**}P <= 0.01$, $^{***}P <= 0.001$, $^{****}P <= 0.0001$). (c) Differences in injured PT cells (Inj PT) between groups in the cortical region (same test as in b). (d) Spatial mapping of the Tnc–Tlr4 ligand–receptor interaction in UIRI kidney sections. (e) FeaturePlot showing the expression pattern of Tlr4 in UIRI kidney.

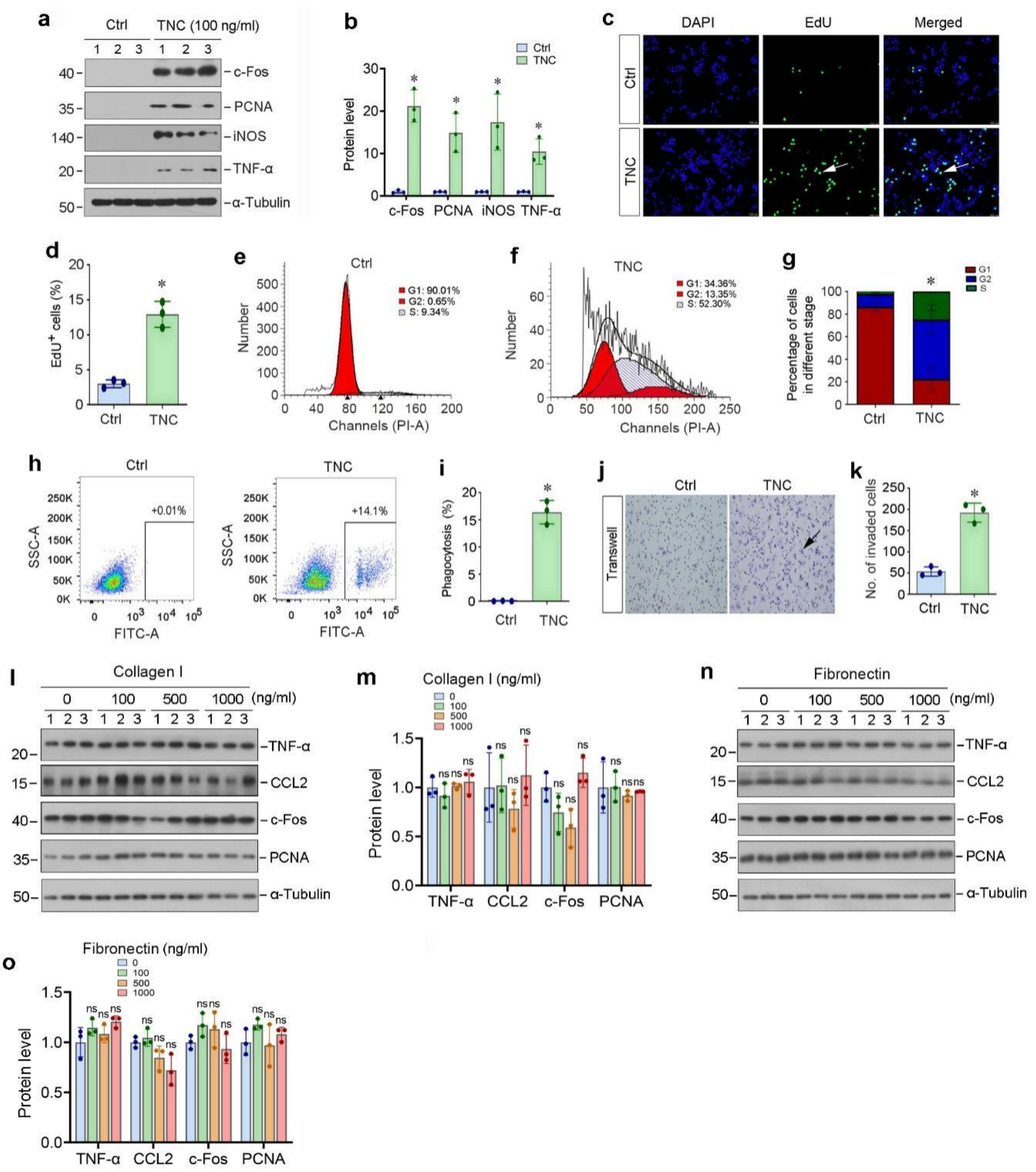


**Figure S5.** TNC induces macrophages proliferation and activation in vitro. (a-b) Western blot analyses show that TNC promoted macrophages proliferation and activation in BMDMs. BMDMs were treated with TNC (100 ng/ml) for 2 days. Representative Western blot (a) and quantitative data (b) are shown. $^{*}P < 0.05$ versus Ctrl (n=3). (c-d) EdU incorporation assay shows that TNC promoted mitogen-mediated DNA synthesis by increasing EdU incorporation in BMDMs. Representative EdU incorporation assay (c) and quantitative data (d) are shown. (e-g) Flow cytometry shows the distribution of different phases in cell cycle in BMDMs after incubation with TNC (100 ng/ml) for 2

days. Representative flow cytometry histograms (e-f) and quantitative data (g) are shown. $^{*}P < 0.05$ versus Ctrl (n=3). (h-i) Flow cytometry shows the macrophage phagocytosis ratio after incubation with TNC (100 ng/ml) for 2 days. Representative flow cytometry histograms (h) and quantitative data (i) are shown. $^{*}P < 0.05$ versus Ctrl (n=3). (j-k) TNC promoted BMDM migration as assessed by Boyden chamber transwell assay. Representative transwell migration assay (j) and quantitative data (k) are presented. $^{*}P < 0.05$ versus Ctrl (n=3). (l-m) Collagen I didn't induce macrophages proliferation and activation. BMDMs were treated with different concentrations of collagen I as indicated for 48 h. Representative Western blot (l) and quantitative data (m) are shown, n=3. (n-o) Fibronectin didn't induce macrophages proliferation and activation. BMDMs were treated with different concentrations of fibronectin as indicated for 48 h. Representative Western blot (n) and quantitative data (o) are shown, n=3.

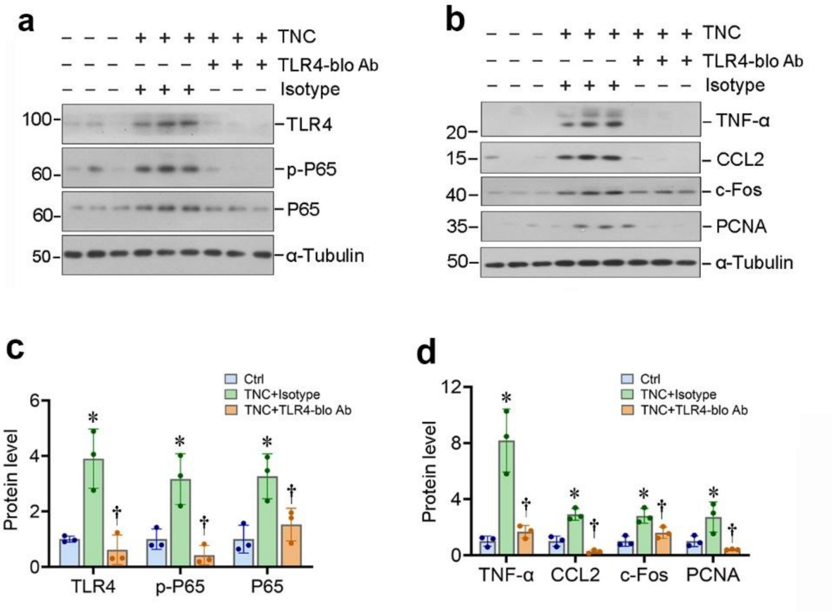


**Figure S6.** Blockade of TLR4 inhibited NF-κB signaling pathway and abolished TNC-induced macrophage proliferation and activation. (a-b) Representative Western blots show that blockade of TLR4 by blocking antibody (TLR4-blo Ab) inhibited TLR4/NF-κB signaling pathway and abolished TNC-induced macrophage proliferation and activation. BMDMs were pretreated with TLR4-blo Ab (5 μg/ml) or isotype IgG (Isotype) for 2 h, then treated with TNC (100 ng/ml) for 48 h. (c-d) Densitometric quantification of TLR4, p-P65, P65, TNF-α, CCL2, c-Fos and PCNA proteins. BMDMs were pretreated with TLR4-blo Ab (5 μg/ml) or isotype IgG (Isotype) for 2 h, then treated with TNC (100 ng/ml) for 48 h. $^{*}P < 0.05$ versus Ctrl, $^{\dagger}P < 0.05$ versus TNC + Isolyte (n=3).

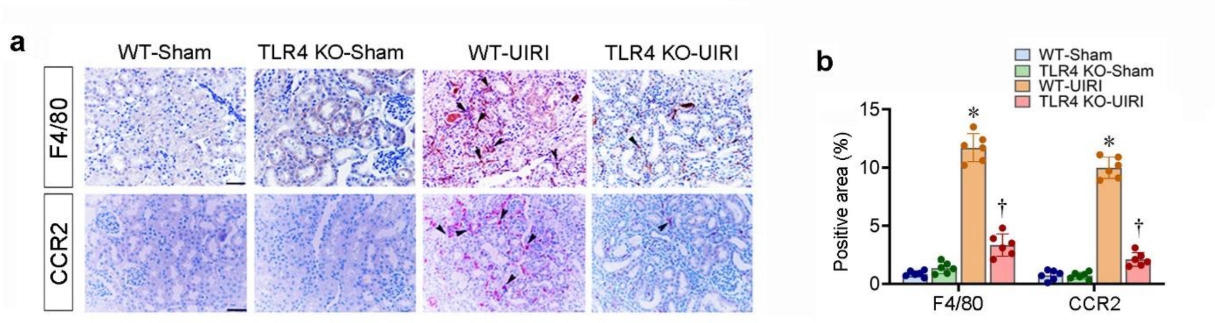


**Figure S7.** Knockout of TLR4 ameliorates renal inflammation in vivo. (a) Representative micrographs show renal expression and localization of F4/80 and CCR2 by immunohistochemical staining in different groups as indicated. (b) Graphic presentation shows the semi-quantitative determination of renal F4/80$^{+}$ and CCR2$^{+}$ area in different groups. At least 10 randomly selected fields were assessed, and results averaged for each kidney. $^{*}P < 0.05$ versus WT-sham, $^{\dagger}P < 0.05$ versus WT-UIRI (n=6).

**Supplementary Figure 8**

Figure 3k

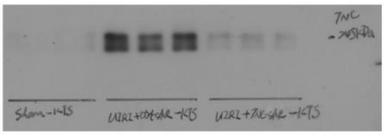

Figure 3m

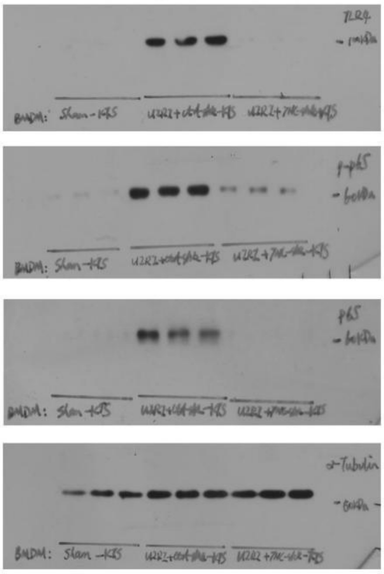

Figure 3o

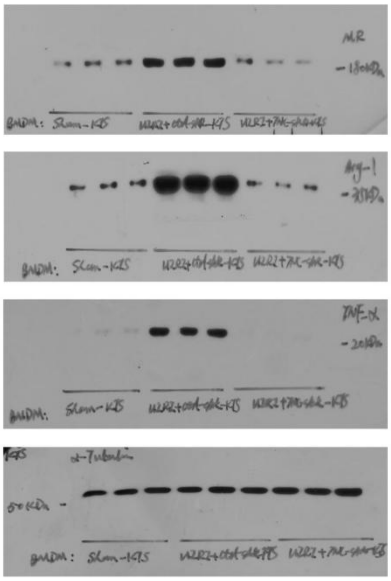

**Supplementary Figure 8**

Figure 4e

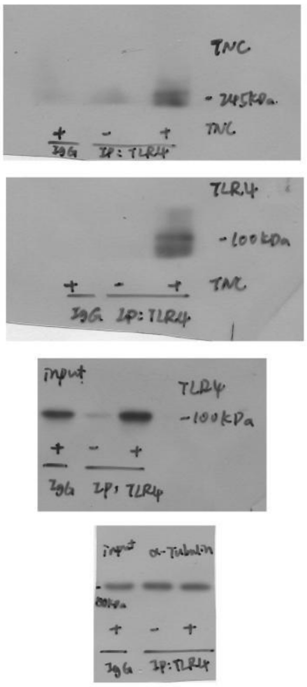


Figure 4f

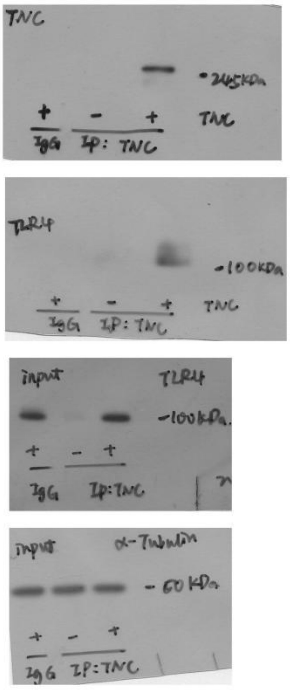


Figure 4g

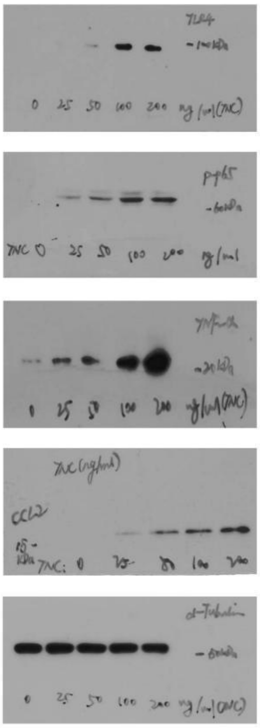


Figure 4i

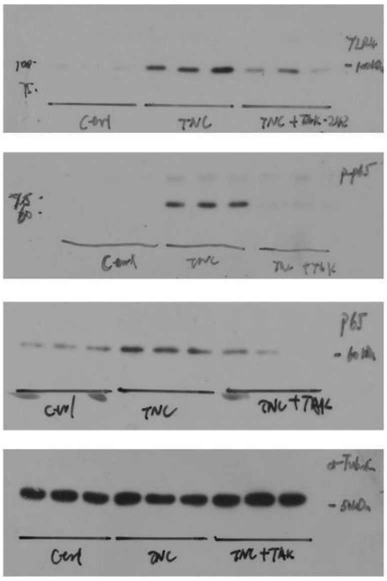


Figure 4k

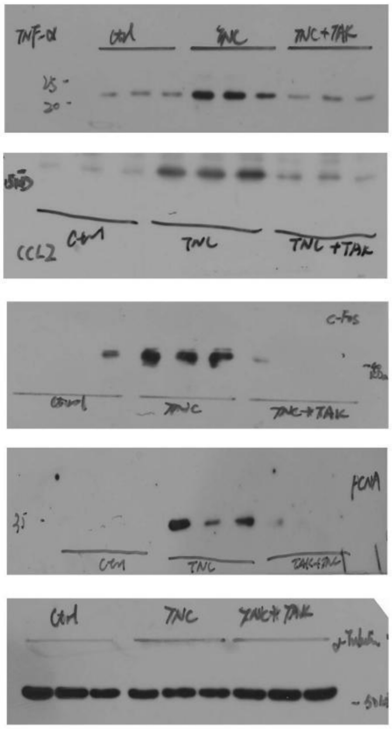

**Supplementary Figure 8**

Figure 5c

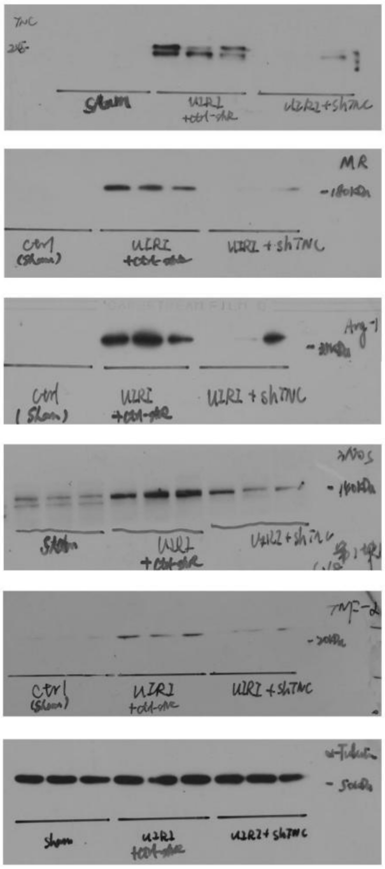

Figure 5f

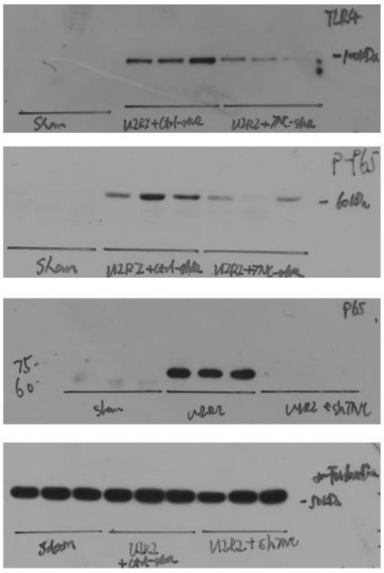

**Supplementary Figure 8**

Figure 6d

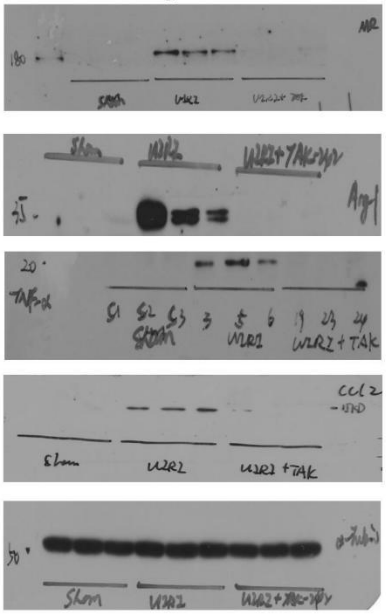

Figure 6f

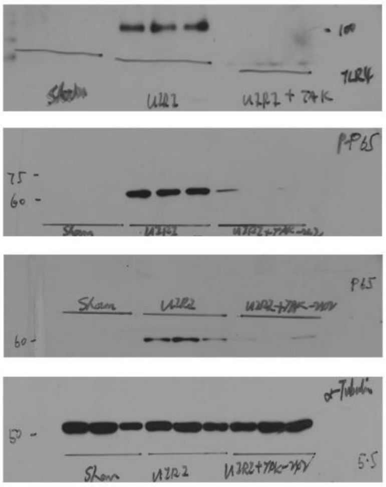

Figure 6h

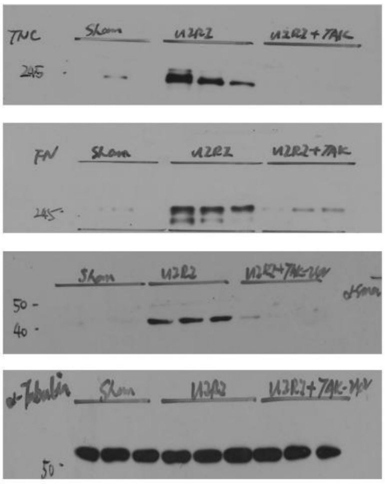

## Supplementary Figure 8

Figure 7a

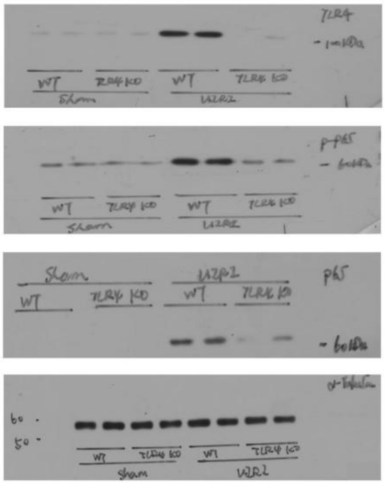

Figure 7i

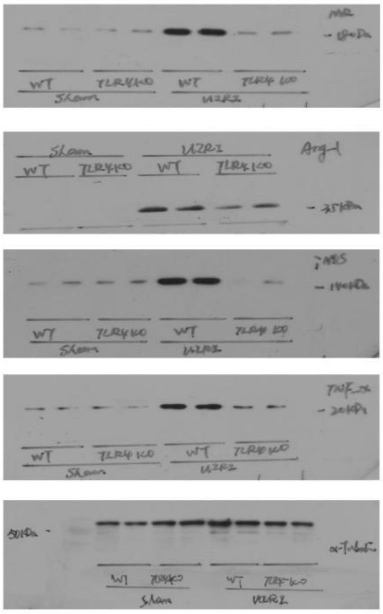

Figure 7k

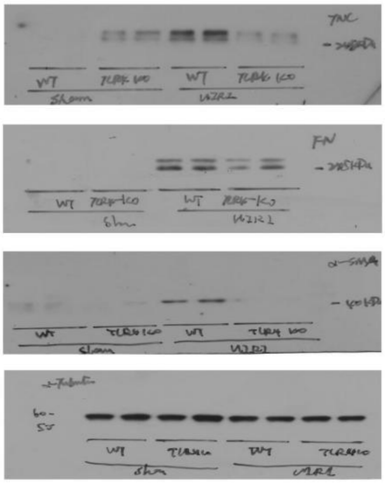

Figure 7q

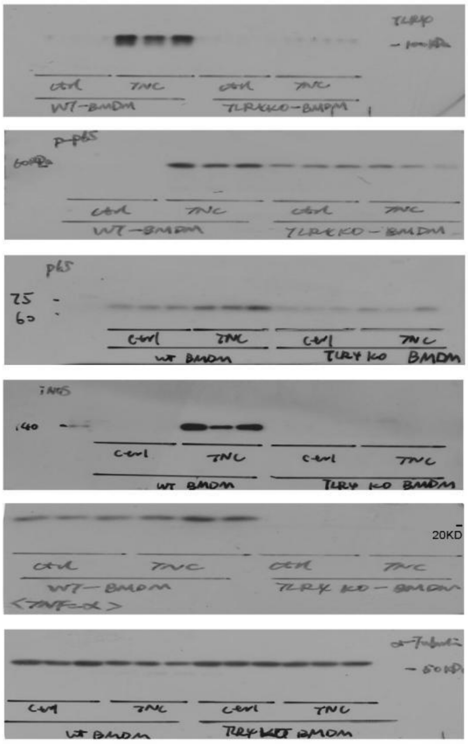

## Supplementary Figure 8

Figure 8b

WT-primer

KO-primer

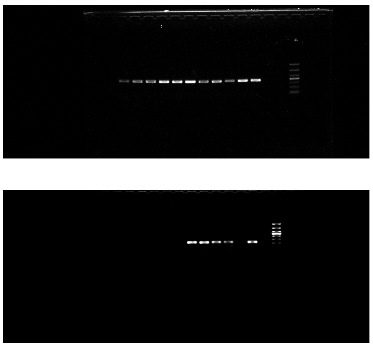

Figure 8e

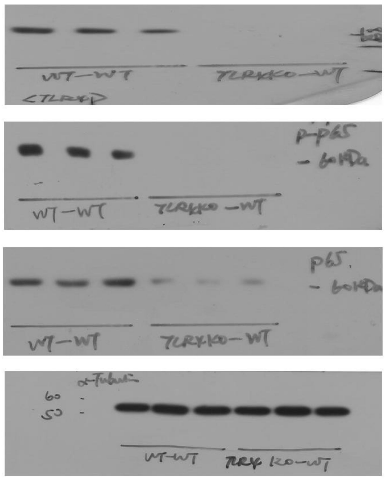

Figure 8j

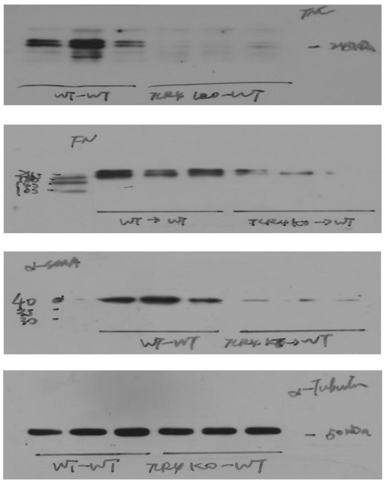

Figure 8h

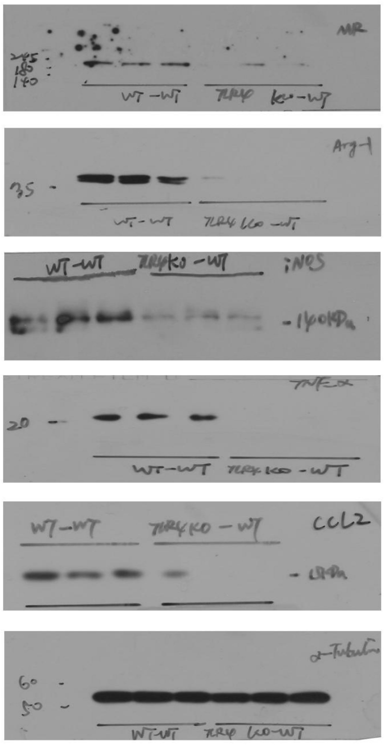

## Supplementary Figure 8

Figure S5a

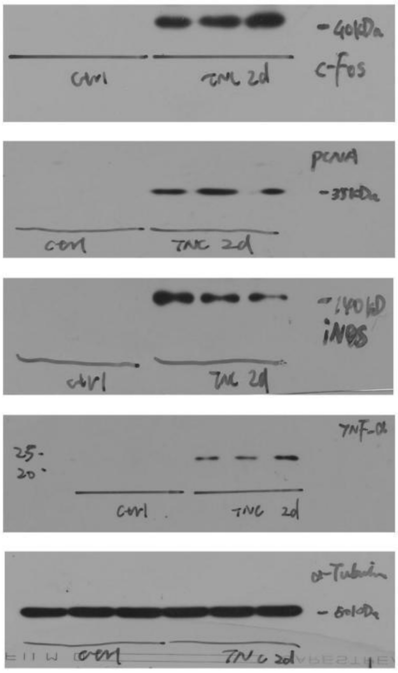


Figure S5l

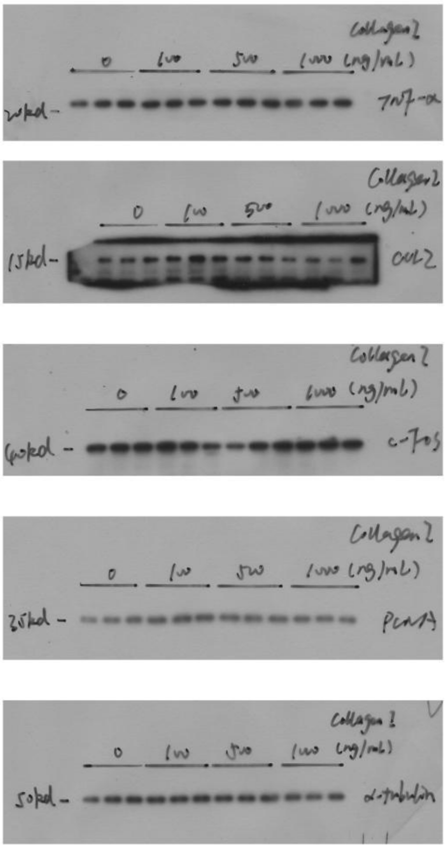


Figure S5n

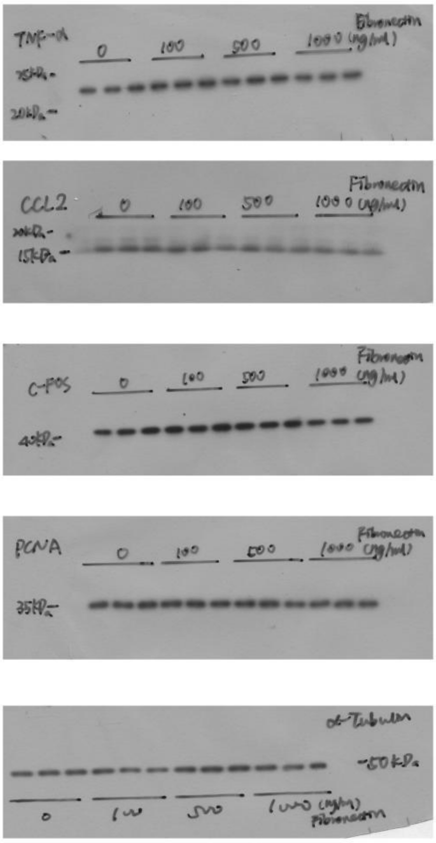

## Supplementary Figure 8

Figure S6a Figure S6b

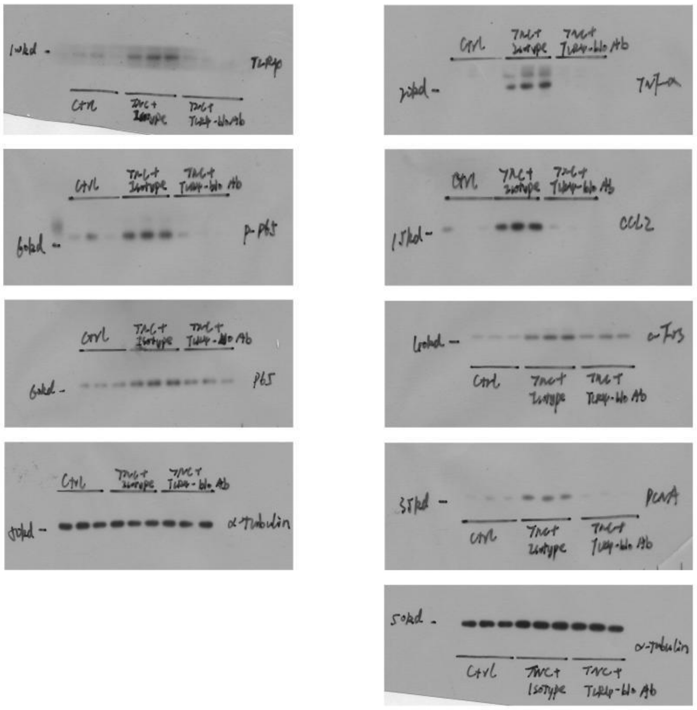

**Figure S8.** Original western blot membranes supporting protein expression analyses in this study
